# Multivitamin Use and Mortality Risk in 3 Prospective US Cohorts

**DOI:** 10.1001/jamanetworkopen.2024.18729

**Published:** 2024-06-26

**Authors:** Erikka Loftfield, Caitlin P. O’Connell, Christian C. Abnet, Barry I. Graubard, Linda M. Liao, Laura E. Beane Freeman, Jonathan N. Hofmann, Neal D. Freedman, Rashmi Sinha

**Affiliations:** 1 Metabolic Epidemiology Branch, Division of Cancer Epidemiology and Genetics, National Cancer Institute, National Institutes of Health, Rockville, Maryland; 2 Biostatistics Branch, Division of Cancer Epidemiology and Genetics, National Cancer Institute, National Institutes of Health, Rockville, Maryland; 3 Occupational and Environmental Epidemiology Branch, Division of Cancer Epidemiology and Genetics, National Cancer Institute, National Institutes of Health, Rockville, Maryland

## Abstract

**Importance:**

One in 3 US adults uses multivitamins (MV), with a primary motivation being disease prevention. In 2022, the US Preventive Services Task Force reviewed data on MV supplementation and mortality from randomized clinical trials and found insufficient evidence for determining benefits or harms owing, in part, to limited follow-up time and external validity.

**Objective:**

To estimate the association of MV use with mortality risk, accounting for confounding by healthy lifestyle and reverse causation whereby individuals in poor health initiate MV use.

**Design, Setting, and Participants:**

This cohort study used data from 3 prospective cohort studies in the US, each with baseline MV use (assessed from 1993 to 2001), and follow-up MV use (assessed from 1998 to 2004), extended duration of follow-up up to 27 years, and extensive characterization of potential confounders. Participants were adults, without a history of cancer or other chronic diseases, who participated in National Institutes of Health–AARP Diet and Health Study (327 732 participants); Prostate, Lung, Colorectal, and Ovarian Cancer Screening Trial (42 732 participants); or Agricultural Health Study (19 660 participants). Data were analyzed from June 2022 to April 2024.

**Exposure:**

Self-reported MV use.

**Main Outcomes and Measures:**

The main outcome was mortality. Cox proportional hazard models were used to estimate hazard ratios (HRs) and 95% CIs.

**Results:**

Among 390 124 participants (median [IQR] age, 61.5 [56.7-66.0] years; 216 202 [55.4%] male), 164 762 deaths occurred during follow-up; 159 692 participants (40.9%) were never smokers, and 157 319 participants (40.3%) were college educated. Among daily MV users, 49.3% and 42.0% were female and college educated, compared with 39.3% and 37.9% among nonusers, respectively. In contrast, 11.0% of daily users, compared with 13.0% of nonusers, were current smokers. MV use was not associated with lower all-cause mortality risk in the first (multivariable-adjusted HR, 1.04; 95% CI, 1.02-1.07) or second (multivariable-adjusted HR, 1.04; 95% CI, 0.99-1.08) halves of follow-up. HRs were similar for major causes of death and time-varying analyses.

**Conclusions and Relevance:**

In this cohort study of US adults, MV use was not associated with a mortality benefit. Still, many US adults report using MV to maintain or improve health.

## Key Points

**Question:**

What is the association between long-term, daily multivitamin use and mortality in generally healthy adults?

**Findings:**

In this cohort study of 390 124 generally healthy adults with more than 20 years of follow-up, daily multivitamin use was not associated with a mortality benefit.

**Meaning:**

These findings suggest that multivitamin use to improve longevity is not supported.

## Teaser

This cohort study examines the association of multivitamin use with mortality risk in 3 large, geographically diverse cohorts of generally healthy US adults.

## Introduction

In the United States, nearly 1 in 3 adults reports recent multivitamin (MV) use.^1^ Prevalence of use is higher among older adults, women, non-Hispanic White individuals, and those with a college education.^1^ Motivations for using MV include to maintain or improve health and prevent chronic disease^2^; consequently, understanding the relationship between MV use and mortality is critically important to public health guidance. In 2022, the US Preventive Services Task Force (USPSTF) reviewed data on MV supplementation and all-cause mortality from randomized clinical trials and found insufficient evidence for determining benefits or harms, owing, in part, to limited follow-up time and lack of external validity.^3^ With decades of follow-up and large populations, prospective cohorts can address these limitations; however, findings from observational studies on MV use and mortality have been mixed,^4,5,6,7,8,9,10,11,12,13,14,15,16,17,18,19,20^ and important issues, which may explain study heterogeneity, need to be systematically addressed. First, confounding by healthy lifestyle is a major concern, as MV users often report eating healthier diets, exercising more, and smoking cigarettes less; this phenomenon has been referred to as the *healthy user effect*.^21^ Second, it is unclear how MV use changes over time and how such changes may affect health. One issue of concern is that patients with diagnosed disease may increase their MV intake because of perceived health benefits; this has been termed the *sick user effect*.^6^

In this study, we investigated the hypothesis that daily MV use is associated with lower mortality risk among generally healthy US adults by leveraging data from 3 large and geographically diverse US cohorts with repeat assessments of MV use and extended follow-up for mortality outcomes. With a combined sample size exceeding 390 000 adults and 164 000 deaths, we aimed to evaluate the association of MV use with the leading causes of chronic disease–related death (ie, cardiovascular disease and cancer) and to systematically explore sources of bias that contribute to uncertainty surrounding the association between MV use and mortality.

## Methods

For this cohort study, the protocol for each included cohort study was approved by the Special Studies Institutional Review Board of the National Cancer Institute; the Prostate, Lung, Colorectal and Ovarian (PLCO) Cancer Screening Trial was also approved by the institutional review board at each of the 10 screening centers. Informed consent was obtained from participants or implied from completion and return of study questionnaires. All data used in this analysis was deidentified. Our study follows the Strengthening the Reporting of Observational Studies in Epidemiology (STROBE) reporting guideline for cohort studies.

### Study Population

Participants were adults in the National Institutes of Health–AARP Diet and Health Study (NIH-AARP) cohort; the PLCO Cancer Screening Trial cohort; and the Agricultural Health Study (AHS) cohort (eFigure 1 in Supplement 1). The NIH-AARP Diet and Health Study began in 1995 to 1996, when questionnaires were mailed to current AARP members who were aged 50 to 71 years and resided in 1 of 2 US metropolitan areas or 6 states.^22^ Overall, 566 398 adults completed the baseline questionnaire. We excluded participants who responded via proxy (15 760 participants); died before questionnaire was received (42 participants); had self-reported or registry-confirmed cancer at baseline (50 727 participants); had self-reported diabetes, myocardial infarction, stroke, or end-stage kidney disease at baseline (105 871 participants); reported extreme caloric intake (4863 participants); or were missing covariates of interest (61 403 participants). The time-varying analysis, which incorporated follow-up questionnaire data collected in 2004, included participants who were censored before the follow-up questionnaire was administered and those who completed a follow-up questionnaire.

The PLCO study was a randomized cancer-screening trial that enrolled 154 887 participants at 10 US centers between 1993 and 2001.^23^ Participants were aged 55 to 74 years at baseline. A dietary questionnaire, which assessed MV use, was administered to the screening group and was considered invalid if the participant died before its completion, had more than 8 missing or multiple-frequency responses, had a missing completion date, or had extreme calorie consumption. We excluded participants who were missing a baseline questionnaire (4918 participants); had a missing or invalid dietary questionnaire (84 392 participants); had cancer (6992 participants) or self-reported a history of diabetes, myocardial infarction, or stroke (9265 participants) at baseline; or were missing covariate data (6588 participants). For the time-varying analysis, we included participants who completed a follow-up dietary history questionnaire approximately 3 years after study entry, beginning in 1998, or who were censored before the dietary history questionnaire.

The AHS enrolled 52 394 licensed pesticide applicators, 4916 commercial pesticide applicators, and 32 345 spouses of private applicators who were aged 18 years or older from Iowa and North Carolina from 1993 to 1997.^24^ Using AHS Data Release Version 202210.00, we excluded participants who did not return a take-home questionnaire (32 019 participants; applicators only); died before receipt of baseline questionnaire (14 participants); were younger than 18 years at enrollment (32 participants); were out-of-state enrollees (257 participants); had self-reported or registry-confirmed cancer at baseline (3404 participants); had self-reported diabetes, myocardial infarction, stroke, or kidney failure at baseline (2772 participants); did not complete a dietary history questionnaire (20 234 participants); had extreme caloric intake (546 participants); or were missing covariate data (10 717 participants). For the time-varying analysis, we included participants who completed a follow-up dietary history questionnaire, from 1999-2003, approximately 5 years after study entry.

Our final analytic sample for the complete-case, pooled analysis included 390 124 participants. The time-varying complete-case analysis included 234 593 of these participants.

### Exposure Assessment

In NIH-AARP, PLCO, and AHS, participants were asked on baseline and follow-up questionnaires about past supplement use. Participants who responded “yes, once per month or more” (NIH-AARP), “yes” (PLCO), or “yes, fairly regularly” (AHS) were asked specifically about frequency of MV use using predefined categories, ranging from never to every day; in subsequent questions, these participants were asked about use of other vitamins and minerals not including MV. Categories of MV use were harmonized across the 3 cohorts such that participants were classified as nonusers, nondaily users, or daily users of MV.

Potential confounders were harmonized across studies and included sex, age, self-reported race and ethnicity, education level, smoking status and intensity, body mass index (BMI; calculated as weight in kilograms divided by height in meters squared), marital status, physical activity level, alcohol intake, coffee intake, Healthy Eating Index 2015 (HEI-2015),^25^ other individual supplement use, and family history of cancer. In NIH-AARP, race and ethnicity were queried in the same question with the following 6 predefined categories: American Indian or Alaskan Native; Asian; Black, not Hispanic; Hispanic; Pacific Islander; and White, not Hispanic. In PLCO, race was queried about using 5 predefined categories (ie, American Indian or Alaskan Native, Asian, Black, Pacific Islander, and White), and Hispanic ethnicity was asked in a separate question. Black and White participants who reported Hispanic ethnicity were classified as Hispanic. In AHS, race was queried about using 5 predefined categories (ie, American Indian or Alaskan Native, Asian or Pacific Islander, Black, White, or other), and Hispanic ethnicity was asked in a separate question. Participants who reported Hispanic ethnicity were classified as Hispanic. To harmonize the race and ethnicity variable across the 3 cohorts, Asian and Pacific Islander categories in NIH-AARP and PLCO were combined to match AHS. Race and ethnicity data were included because these data were assessed in each of the 3 cohorts as part of the collection of demographic data.

### Mortality Ascertainment

Participants were followed from baseline MV assessment until date of death, loss to follow-up, or the end of the study period (NIH-AARP and AHS: December 31, 2019; PLCO: December 31, 2020). Death was ascertained through the National Death Index. Cause-specific mortality was ascertained from the *International Classification of Diseases, Ninth Revision (ICD-9)* or *International Statistical Classification of Diseases and Related Health Problems, Tenth Revision (ICD-10)* code for underlying cause of death from death certificates. Causes of death were defined as cancer (*ICD-9* codes: 140-208, 238.6; *ICD-10* codes: C00-C97), heart disease (*ICD-9* codes: 390-398, 402, 404, 410-429; *ICD-10* codes: I00-I09, I11, I13, I20-I51), and cerebrovascular diseases (*ICD-9* codes: 430-438; *ICD-10* codes: I60-I69).^26^ For cause-specific mortality analyses, proportional hazards models were fit separately for each type of cause-specific mortality, and persons who died from other causes were censored at their date of death.^27^

### Statistical Analysis

We conducted individual study and pooled analyses. Baseline results from the 3 studies were similar and meta-analysis indicated low between-study heterogeneity. Therefore, pooled analyses are presented as the main results. Owing to study differences in covariate assessment during follow-up, time-varying analyses were run in each cohort, and risk estimates were meta-analyzed.

First, we tabulated demographic and lifestyle factors by MV use and assessed differences for categorical and continuous variables using χ^2^ and Kruskal-Wallis tests, respectively. We used Cox proportional hazard regression models to estimate hazard ratios (HRs) and 95% CIs for the associations of nondaily and daily MV use, with nonusers as the reference group, with mortality risk. Time since baseline was used as the underlying time metric. We tested the proportional hazard assumption using the cox.zph function in the survival package in R statistical software version 4.2.2 (R Project for Statistical Computing). Due to violation of this assumption (*P* < .001), we estimated relative risks in the first and second halves of follow-up (ie, follow-up period 1 [FP1] and follow-up period 2 [FP2], respectively) by including a binary variable for follow-up period, defined based on the midpoint of study follow-up, and the interaction term between this variable and MV use in all models.

We ran age- and sex-adjusted models and further adjusted for race and ethnicity, education, marital status, BMI, cigarette smoking, daily alcohol intake, daily coffee intake, HEI-2015, family history of cancer, and individual supplement use. All covariates, except for family history of cancer, changed at least 1 of the pooled risk coefficients by more than 10%. Multivariable-adjusted models were stratified by potential modifiers; modification was evaluated using the likelihood ratio test comparing models with and without an interaction term between the variable of interest and MV use. To assess potential bias due to unmeasured, underlying health conditions, we estimated HRs for MV use and all-cause mortality in the first 5 years, 5 to 10 years, and 10 or more years after baseline MV assessment.

In time-varying analyses, we incorporated MV use assessed approximately 9, 3, and 5 years after baseline in NIH-AARP, PLCO, and AHS, respectively. In NIH-AARP, BMI, smoking, physical activity, individual supplement use, and family history of cancer were reassessed. In PLCO, daily alcohol intake, coffee intake, HEI-2015 quartile, and individual supplement use were reassessed. In AHS, smoking, BMI, daily alcohol intake, and individual supplement use were reassessed. The survival package in R was used to run a Cox proportional hazard regression model with a time-dependent variable for MV use. Other covariates were updated where possible; otherwise, baseline data were kept constant. The R function tmerge was used to create the time-dependent dataset for these analyses. The results from the individual study time-varying analyses were meta-analyzed using a fixed-effects model. The metafor package in R was used for the meta-analysis. A 2-sided *P* < .05 was considered statistically significant. Data were analyzed from June 2022 to April 2024.

## Results

In our analytic sample of 390 124 participants (median [IQR] age, 61.5 [56.7-66.0] years; 216 202 [55.4%] male), including 327 732 from NIH-AARP, 42 732 from PLCO, and 19 660 from AHS, there were 7 861 485 person-years of follow-up (NIH-AARP: 6 576 546 person-years; PLCO: 827 313 person-years; AHS: 457 626 person-years). A total of 164 762 participants died over the study period, with 49 836 deaths attributed to cancer, 35 060 deaths attributed to heart diseases, and 9275 attributed to cerebrovascular diseases (Table 1). Participants in the AHS were younger (median [IQR] age, 47.0 [38.0-56.0] years) than participants in NIH-AARP (median [IQR] age, 61.9 [57.2-66.1] years) and PLCO (median [IQR] age, 62.0 [58.0-66.0] years), but, in each of the 3 cohorts, median age was similar for daily MV users and nonusers (Table 2). Among daily MV users, 49.3% and 42.0% were female and college educated, compared with 39.3% and 37.9% among nonusers, respectively. In contrast, 11.0% of daily users, compared with 13.0% of nonusers, were current smokers. Participants who used MV, compared with those who did not, were also more likely to use individual supplements and have lower BMI and better diet quality (Table 2). Daily MV users were less likely than nonusers to be currently married in NIH-AARP and PLCO but more likely to be married in AHS, likely owing to different age distributions. MV use did not vary meaningfully by race or ethnicity or family history of cancer (Table 2; eTable 1 in Supplement 1).

**Table 1.** Participants, Follow-Up Time, and Deaths in the 3 Cohorts

Characteristic	No.			
	AARP	PLCO	AHS	Total
Participants in study	327 732	42 732	19 660	390 124
Follow-up, person-years	6 576 546	827 313	457 626	7 861 485
Follow-up, median (IQR), y	23.5 (17.9-23.6)	21.3 (15.7-23.5)	23.9 (23.0-25.2)	23.5 (18.0-23.6)
All-cause mortality	145 632	15 898	3232	164 762
Diseases of the heart mortality	31 135	3221	704	35 060
Cancer mortality	44 197	4605	1034	49 836
Cerebrovascular diseases mortality	8143	945	187	9275

Abbreviations: AHS, Agricultural Health Study; PLCO, Prostate, Lung, Colorectal and Ovarian cancer cohort.

**Table 2.** Baseline Characteristics of Study Participants by Cohort, According to Multivitamin Use^a^

Characteristic	Participants, No. (%)					
	AARP (n = 327 732)		PLCO (n = 42 732)		AHS (n = 19 660)	
	Nonuse (n = 144 947)	Daily use (n = 140 956)	Nonuse (n = 19 353)	Daily use (n = 18 908)	Nonuse (n = 15 366)	Daily use (n = 3443)
Age, median (IQR), y	62.0 (57.2-66.2)	62.2 (57.4-66.3)	62.0 (58.0-66.0)	62.0 (58.0-66.0)	47.0 (39.0-56.0)	48.0 (38.0-57.0)
Sex						
Male	89 804 (62.0)	74 341 (52.7)	11 166 (57.7)	7497 (39.6)	8068 (52.5)	953 (27.7)
Female	55 143 (38.0)	66 615 (47.3)	8187 (42.3)	11 414 (60.4)	7298 (47.5)	2490 (72.3)
Race and ethnicity						
American Indian or Alaska Native	362 (0.2)	324 (0.2)	33 (0.2)	36 (0.2)	22 (0.1)	3 (0.1)
Asian or Pacific Islander	1897 (1.3)	1832 (2.8)	599 (3.1)	674 (3.6)	4 (0.0)	1 (0.0)
Hispanic	2601 (1.8)	2371 (1.7)	241 (1.2)	267 (1.4)	115 (0.7)	19 (0.6)
Non-Hispanic Black	5101 (3.5)	3931 (1.3)	676 (3.5)	500 (2.6)	66 (0.4)	9 (0.3)
Non-Hispanic White	134 986 (93.1)	132 498 (94.0)	17 804 (92.0)	17 431 (92.2)	15 159 (98.7)	3411 (99.1)
College educated	57 565 (39.7)	60 572 (43.0)	6937 (35.8)	7175 (37.9)	3530 (23.0)	881 (25.6)
Current smoker	19 689 (13.6)	16 081 (11.4)	2062 (10.7)	1624 (8.6)	1649 (10.7)	221 (6.4)
High physical activity	63 541 (43.8)	71 959 (51.1)	7082 (36.6)	7993 (42.3)	6488 (42.2)	1571 (45.6)
Individual supplement use	50 977 (35.2)	119 735 (84.9)	6554 (33.9)	15 398 (81.4)	5044 (32.8)	2037 (59.2)
Alcohol intake, 0 drinks/d	32 001 (22.1)	30 147 (21.4)	3810 (19.7)	4188 (22.1)	5504 (35.8)	1310 (38.0)
Coffee intake, 0 cups/d	15 380 (10.6)	15 509 (11.0)	2106 (10.9)	2398 (12.7)	4543 (29.6)	1086 (31.5)
BMI, median (IQR)	26.4 (23.9-29.3)	25.8 (23.4-28.6)	26.6 (24.3-29.6)	25.9 (23.6-29.1)	26.4 (23.7-29.3)	25.3 (22.7-28.3)
HEI-2015 score, median (IQR)	67.1 (59.8-73.5)	69.9 (63.0-75.7)	65.6 (59.2-71.6)	68.7 (62.6-74.2)	60.9 (54.5-67.2)	64.1 (57.9-70.4)
Currently married or living as married	104 174 (71.9)	92 912 (65.9)	15 757 (81.4)	14 455 (76.4)	14 262 (92.8)	3296 (95.7)
Family history of cancer	74 673 (51.5)	73 402 (52.1)	10 798 (55.8)	10 760 (56.9)	7431 (48.4)	1720 (50.0)

Abbreviations: AHS, Agricultural Health Study; BMI, body mass index (calculated as weight in kilograms divided by height in meters squared); HEI-2015, Healthy Eating Index 2015; PLCO, Prostate, Lung, Colorectal and Ovarian cancer cohort.

^a^ All exposures were associated with multivitamin use, with *P* < .001.

In the pooled analysis, daily MV users had a higher mortality risk than nonusers (FP1: HR, 1.04; 95% CI, 1.02-1.07; FP2: HR, 1.04; 95% CI, 0.99-1.08) (Table 3). However, HR estimates were close to 1.0 for risk of all-cause, heart disease, cancer, and cerebrovascular disease mortality (Table 3; eTable 2 in Supplement 1).

**Table 3.** Pooled HRs Between Multivitamin Use and Mortality

Cause of death	Follow-up period 1^a^		Follow-up period 2^a^		*P* value
	Nondaily use	Daily use	Nondaily use	Daily use	
**All-cause mortality**						
Deaths, No.	4505	17 843	13 822	52 834	NA
Age and sex-adjusted HR (95% CI)	1.06 (1.02-1.09)	0.99 (0.97-1.01)	0.92 (0.86-0.99)	0.99 (0.95-1.04)	<.001
Multivariable-adjusted HR (95% CI)^b^	1.09 (1.05-1.13)	1.04 (1.02-1.07)	0.95 (0.88-1.02)	1.04 (0.99-1.08)	<.001
**Heart disease mortality**						
Deaths, No.	789	3422	2930	11 609	NA
Age and sex-adjusted HR (95% CI)	0.99 (0.91-1.07)	1.00 (0.95-1.05)	0.90 (0.77-1.06)	1.00 (0.90-1.10)	.95
Multivariable-adjusted HR (95% CI)^b^	1.03 (0.95-1.11)	1.06 (1.01-1.11)	0.93 (0.79-1.10)	1.04 (0.94-1.15)	.06
**Cancer mortality**						
Deaths, No.	1937	7426	3748	13 221	NA
Age and sex-adjusted HR (95% CI)	1.02 (0.97-1.07)	0.96 (0.93-0.99)	0.90 (0.81-1.01)	0.94 (0.87-1.01)	.004
Multivariable-adjusted HR (95% CI)^b^	1.05 (1.00-1.10)	1.01 (0.98-1.05)	0.93 (0.84-1.04)	0.98 (0.91-1.06)	.20
**Cerebrovascular diseases mortality**						
Deaths, No.	186	808	839	3370	NA
Age and sex-adjusted HR (95% CI)	1.06 (0.90-1.24)	1.05 (0.95-1.16)	0.97 (0.70-1.36)	1.07 (0.87-1.31)	.58
Multivariable-adjusted HR (95% CI)^b^	1.04 (0.89-1.23)	1.05 (0.95-1.16)	0.97 (0.69-1.36)	1.06 (0.86-1.31)	.66

Abbreviations: HR, hazard ratio; NA, not applicable.

^a^ The proportional hazards assumption was violated (*P* < .001). Therefore, follow-up time was stratified by the midpoint, and HRs were calculated using an interaction term between follow-up period and the exposure variable. Follow-up period 1 was the first 12 years of follow-up and follow-up period 2 was the last 15 years of follow-up.

^b^ Models were adjusted sex (male or female), age at enrollment (years), race and ethnicity (American Indian or Alaska Native, Asian or Pacific Islander, Hispanic, non-Hispanic Black, or non-Hispanic White), education (≤11 years; 12 years, completed high school or General Educational Development; post–high school training; some college; college and postgraduate; or other), body mass index (calculated as weight in kilograms divided by height in meters squared) category (<18.5, 18.5 to <25, 25 to <30, ≥30), marital status (married or living as married, divorced or separated, widowed, or never married), smoking status (never smoker, former smoker, current smoker ≤20 cigarettes/d, current smoker 21-40 cigarettes/d, or current smoker >40 cigarettes/d), alcohol consumption (0 drinks per day, <1 drink/d, 1 to <2 drinks/d, 2 to <3 drinks/d, ≥3 drinks/d), physical activity level (never, low, moderate, high), coffee intake (0 cups/d, <1 cup/d, 1 cup/d, 2-3 cups/d, 4-5 cups/d, or ≥6 cups/d), family history of cancer (yes or no), Healthy Eating Index 2015 quartile (quartile 1, 21.55 to <60.90; quartile 2, 60.90 to <68.00; quartile 3, 68:00 to <74.20; and quartile 4, 74.20 to <96.10), and use of individual supplements (yes or no). Detailed results for each cohort are presented in eTable 2 in Supplement 1.

**Figure 1.**
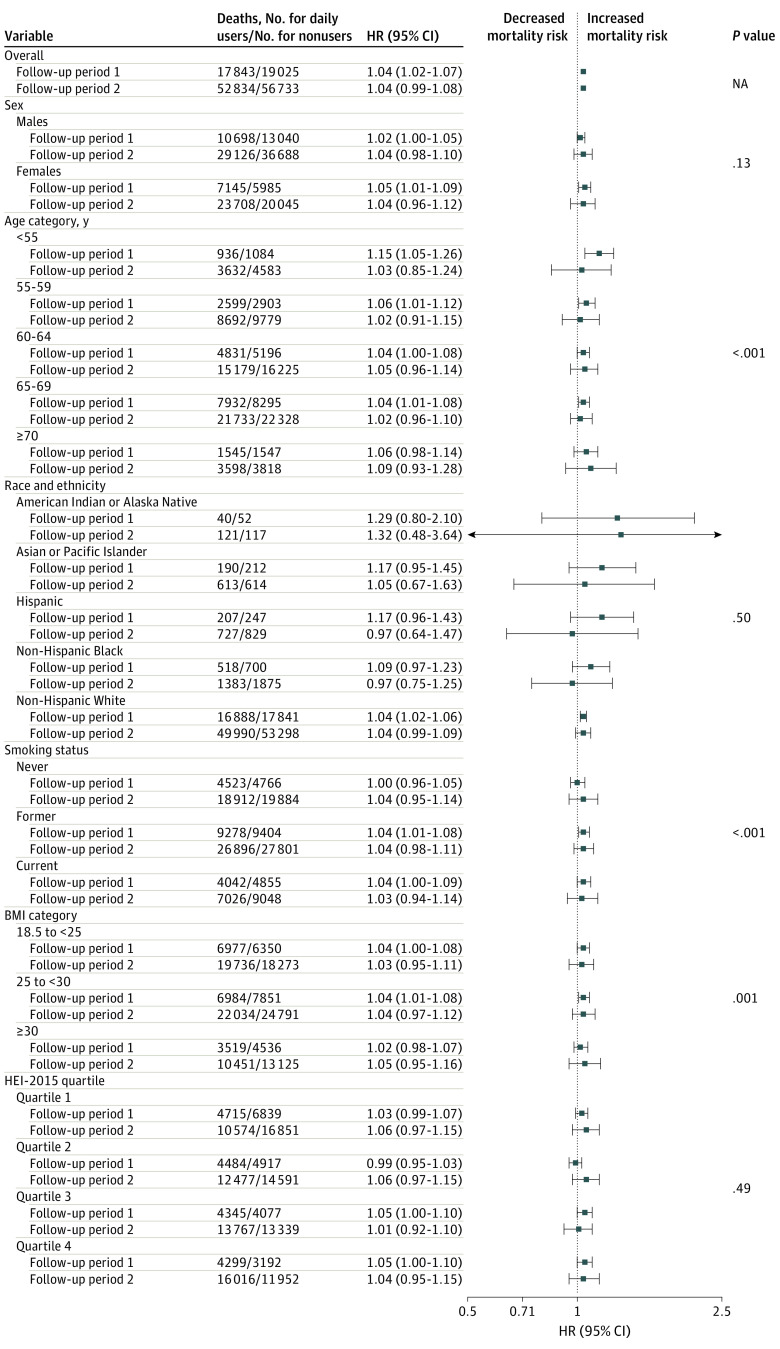
Stratified Baseline Estimates for the Association of Daily Multivitamin Use and All-Cause Mortality (N = 390 124) The proportional hazards assumption was violated (*P* < .001). Therefore, follow-up time was stratified by the midpoint, and hazard ratios (HRs) were calculated using an interaction term between follow-up period and the exposure variable. Follow-up period 1 was the first 12 years of follow-up, and follow-up period 2 was the last 15 years of follow-up. *P* value represents the significance of the likelihood ratio test of each effect modifier. Models were stratified by study and adjusted for sex (male or female), age at enrollment (years), race and ethnicity, education (≤11 years; 12 years, completed high school or General Educational Development; post–high school training; some college; college and postgraduate; or other), body mass index (BMI; calculated as weight in kilograms divided by height in meters squared) category, marital status (married or living as married, divorced or separated, widowed, or never married), smoking status (never smoker, former smoker, current smoker ≤20 cigarettes/d, current smoker 21-40 cigarettes/d, or current smoker >40 cigarettes/d), alcohol consumption (0 drinks per day, <1 drink/d, 1 to <2 drinks/d, 2 to <3 drinks/d, ≥3 drinks/d), physical activity level (never, low, moderate, high), coffee intake (0 cups/d, <1 cup/d, 1 cup/d, 2-3 cups/d, 4-5 cups/d, or ≥6 cups/d), family history of cancer (yes or no), Healthy Eating Index 2015 (HEI-2015) quartile (quartile 1, 21.55 to <60.90; quartile 2, 60.90 to <68.00; quartile 3, 68:00 to <74.20; quartile 4, 74.20 to <96.10), and use of individual supplements (yes or no). NA indicates not applicable.

We observed potential qualitative effect modification by age, smoking status, and BMI, but not by sex, race and ethnicity, or HEI-2015. In the FP1, HR estimates for daily MV use and all-cause mortality were higher for the youngest (<55 years) age group (HR, 1.15; 95% CI, 1.05-1.26); HR estimates for daily MV vs nonusers were similar by smoking and BMI status (Figure 1) but varied for nondaily MV vs nonusers (eFigure 2 in Supplement 1), such that in FP1, HR estimates for nondaily MV use and all-cause mortality were higher for former (HR, 1.10; 95% CI, 1.05-1.16) and current (HR, 1.09; 95% CI, 1.02-1.16) smokers as well as for individuals with a BMI in the normal range (HR, 1.10; 95% CI, 1.09-1.22).

**Figure 2.**
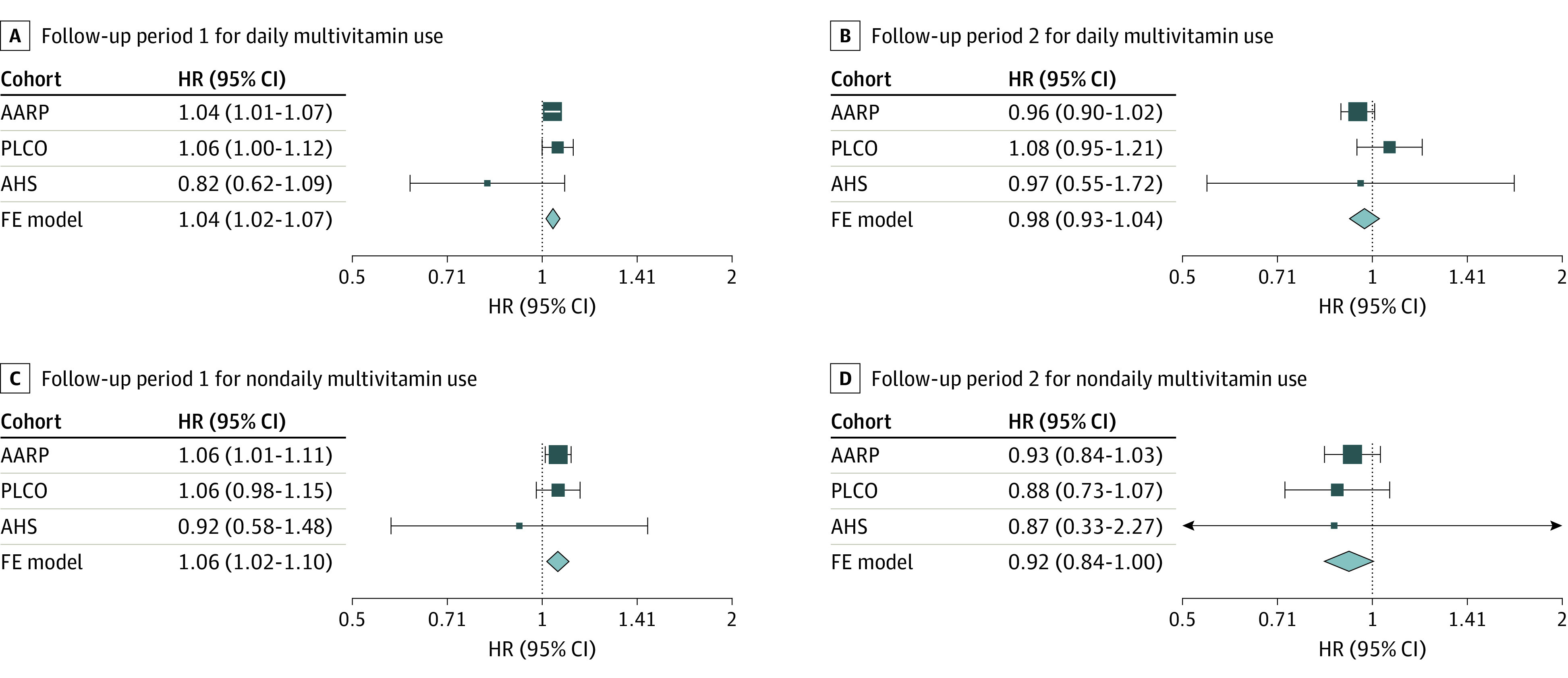
Meta-Analysis of the Time-Varying Estimates for the Association of Multivitamin Use and All-Cause Mortality The proportional hazards assumption was violated (*P* < .001). Therefore, follow-up time was stratified by the midpoint, and hazard ratios (HRs) were calculated using an interaction term between follow-up period and the exposure variable. Maximum follow-up time was 24 years for the AARP cohort (172 496 participants; 78 523 deaths), 27 years for the Prostate, Lung, Colorectal and Ovarian (PLCO) cancer cohort (42 732 participants; 15 898 deaths), and 26 years for the Agricultural Health Study (AHS) cohort (19 365 participants; 3149 deaths); follow-up period 1 was the first 12 years of follow-up and follow-up period 2 was the last 15 years of follow-up. Models were adjusted sex (male or female), age at enrollment (years), race and ethnicity (American Indian or Alaska Native, Asian or Pacific Islander, Hispanic, non-Hispanic Black, or non-Hispanic White), education (≤11 years; 12 years, completed high school or General Educational Development; post–high school training; some college; college and postgraduate; or other), body mass index (calculated as weight in kilograms divided by height in meters squared) category (<18.5, 18.5 to <25, 25 to <30, ≥30), marital status (married or living as married, divorced or separated, widowed, or never married), smoking status (never smoker, former smoker, current smoker ≤20 cigarettes/d, current smoker 21-40 cigarettes/d, or current smoker >40 cigarettes/d), alcohol consumption (0 drinks per day, <1 drink/d, 1 to <2 drinks/d, 2 to <3 drinks/d, ≥3 drinks/d), physical activity level (never, low, moderate, high), coffee intake (0 cups/d, <1 cup/d, 1 cup/d, 2-3 cups/d, 4-5 cups/d, or ≥6 cups/d), family history of cancer (yes or no), Healthy Eating Index 2015 quartile (NIH-AARP: quartile 1, 21.5 to <61.5; quartile 2, 61.5 to <68.6; quartile 3, 68.6 to <74.7; quartile 4, 74.7 to 98; PLCO: quartile 1, 28.5 to <60.8; quartile 2, 60.8 to <67.3; quartile 3, 67.3 to <73.1; quartile 4, 73.1 to 95; AHS: quartile 1, 21.9 to <55.3; quartile 2, 55.3 to <61.8; quartile 3, 61.8 to <68.2; quartile 4, 68.2 to 95), and use of individual supplements (yes or no).

Time-varying analyses included 234 593 participants (eTable 3 in Supplement 1). HR estimates were similar in the NIH-AARP and PLCO cohorts in FP1 (NIH-AARP: HR, 1.04; 95% CI, 1.01-1.07; PLCO: HR, 1.06; 95% CI, 1.00-1.12) with a higher mortality risk for daily MV users compared with nonusers (Figure 2). In FP2, the HR for NIH-AARP, the largest of the 3 studies, was attenuated and the 95% CI included 1.00. In the meta-analysis incorporating the time-varying estimates from all 3 cohorts, daily MV use, compared with nonuse, was associated with a 4% higher risk of all-cause mortality in FP1 (HR, 1.04; 95% CI, 1.02-1.07) but not in FP2 (HR, 0.98; 95% CI, 0.93-1.04) (Figure 2).

## Discussion

In this cohort study of 390 124 generally healthy US adults with more than 20 years of follow-up, daily MV use was not associated with a mortality benefit. In contrast, we found that daily MV use vs nonuse was associated with 4% higher mortality risk. The results of the time-varying analysis, incorporating a second MV use assessment, were consistent with the pooled baseline estimates and support our conclusion of no mortality benefit. Finally, by pooling data from 3 large cohorts, we could explore heterogeneity across key population subgroups, including understudied sociodemographic subgroups, which was identified as a research gap in the 2022 USPSTF review.^3^ In stratified analyses, we found no evidence of effect modification by race and ethnicity, education, or diet quality.

In the US, MV use declined by 6% from 1999 to 2011 but remains popular, with nearly 1 in 3 adults reporting recent use.^1,2^ This downward trend may, in part, reflect growing uncertainty about the effectiveness of MV supplementation for preventing disease, following the publication of several studies that reported no benefit of MV use for reducing risk of cardiovascular disease, cancer, or mortality.^6,13,20^ In 2014, the USPSTF concluded that “current evidence is insufficient to assess the balance of benefits and harms of the use of multivitamins for the prevention of cardiovascular disease or cancer,”^28^ and in 2022, after conducting a pooled analysis of 9 randomized clinical trials, the USPSTF conclusion remained largely the same, stating that “the evidence is insufficient to determine the balance of benefits and harms of supplementation with multivitamins for the prevention of cardiovascular disease or cancer.”^3^ However, in line with our findings, 1 of the included studies, the Physicians’ Health Study II, which was a large randomized double-blind, placebo-controlled trial of a daily MV use, observed no benefit for reducing cardiovascular disease or mortality in male physicians despite more than a decade of treatment and follow-up.^29^

Prospective cohort studies have also been inconsistent. A few studies have found no benefit of MV use for reducing cardiovascular disease, cancer, or mortality.^5,6,7,8,13,20,30^ Others have found potential benefit for daily MV use and cardiovascular disease mortality,^9,12^ and 1 study in a nationally representative sample of US adults with approximately 20 years of follow-up found that women who reported use of multivitamin or multimineral products for more than 3 years, compared with those who did not, had a lower risk of cardiovascular disease mortality.^19^ Yet, other studies have reported adverse associations for MV use and mortality among older women^14^ and for prostate cancer mortality among men.^15,31^

Varying results across observational studies may be explained, in part, by differences in MV composition or by confounding.^32^ For example, MV users may be more health conscious than nonusers; this could translate into healthier diets, more frequent engagement in physical activity and preventative care, or lower rates of obesity and smoking. Confounding by healthy lifestyle would likely result in spurious inverse associations. Conversely, it could be argued that those who are sick or older than 65 years are more likely to initiate MV use. This phenomenon could result in a noncausal positive association, since these individuals have a higher risk of mortality than their healthier or younger counterparts. Furthermore, baseline measures do not account for the possibility of change in MV use over time.

Strengths of our study are highlighted by how we addressed these concerns. First, we harmonized and pooled complete data from individuals who participated in 1 of 3 large cohorts that collected detailed information on demographics and lifestyle factors and were therefore able to evaluate potential differences in relative risk by demographic and lifestyle factors. Additionally, with the extended follow-up periods of 24 years in NIH-AARP, 27 years in PLCO, and 26 years in AHS and repeated assessments of MV use, we were better able evaluate the long-term association of daily MV use with mortality risk.

### Limitations

Our study has some limitations. First, it is an observational study and residual confounding by poorly measured or unmeasured confounders (eg, health care utilization) may bias risk estimates. However, we excluded individuals with a history of cancer and other chronic disease at baseline and those with missing data; additionally, we carefully adjusted for major mortality risk factors and, where possible, updated variables, like smoking and BMI, in time-varying analyses. Second, there is the possibility for nondifferential exposure misclassification owing to faulty memory of sporadic MV usage. For this reason, we focused our interpretation on daily use vs nonuse. Additionally, the prospective nature of the study mitigates the potential for differential exposure misclassification. Third, selection bias is possible as the participants with missing data could be systematically different than those with complete data. However, age- and sex-adjusted HR estimates from the complete case analytic sample and the larger sample with missing covariate data were similar. Nevertheless, because of these exclusions, generalizability to the total US population may be limited. Fourth, the 3 studies include mostly White individuals, but pooling across the 3 studies improved statistical power for subgroup analyses. Lastly, we cannot assess latency of the association of MV use and the cumulative association over the life span.

## Conclusions

In this cohort study of 390 124 US adults without a history of major chronic diseases, we did not find evidence to support improved longevity among healthy adults who regularly take multivitamins. However, we cannot preclude the possibility that daily MV use may be associated with other health outcomes related to aging.^33^

## Notes

Supplement 1. **eFigure 1.** Flowchart for Final Analytic Sample **eTable 1.** Baseline Characteristics of Study Participants by Cohort, According to Multivitamin Use **eTable 2.** Study-Specific Hazard Ratios Between Multivitamin Use and Mortality **eFigure 2.** Forest Plot of Stratified Baseline Estimates for the Association of Nondaily Multivitamin Use and All-Cause Mortality **eTable 3.** Baseline Characteristics of Time-Varying Analysis Participants by Cohort, According to Multivitamin Use

Supplement 2.
Data Sharing Statement
