# Supplementary material for: Multivitamin Use and Mortality Risk in 3 Prospective US Cohorts
Source: JAMA Netw Open. 2024 Jun 26;7(6):e2418729. doi: 10.1001/jamanetworkopen.2024.18729 (PMC11208972; doi:10.1001/jamanetworkopen.2024.18729)
Supplement: Supplement 1. — eFigure 1. Flowchart for Final Analytic Sample eTable 1. Baseline Characteristics of Study Participants by Cohort, According to Multivitamin Use eTable 2. Study-Specific Hazard Ratios Between Multivitamin Use and Mortality eFigure 2. Forest Plot of Stratified Baseline Estimates for the Association of Nondaily Multivitamin Use and All-Cause Mortality eTable 3. Baseline Characteristics of Time-Varying Analysis Participants by Cohort, According to Multivitamin Use [file jamanetwopen-e2418729-s001.pdf]

# Supplemental Online Content

Loftfield E, O'Connell CP, Abnet CC, et al. Multivitamin use and mortality risk in 3 prospective US cohorts. *JAMA Netw Open*. 2024;7(6):e2418729. doi:10.1001/jamanetworkopen.2024.18729


**eFigure 1.** Flowchart for Final Analytic Sample

**eTable 1.** Baseline Characteristics of Study Participants by Cohort, According to Multivitamin Use

**eTable 2.** Study-Specific Hazard Ratios Between Multivitamin Use and Mortality

**eFigure 2.** Forest Plot of Stratified Baseline Estimates for the Association of Nondaily Multivitamin Use and All-Cause Mortality

**eTable 3.** Baseline Characteristics of Time-Varying Analysis Participants by Cohort, According to Multivitamin Use


This supplemental material has been provided by the authors to give readers additional information about their work.

© 2024 Loftfield E et al. *JAMA Network Open.*

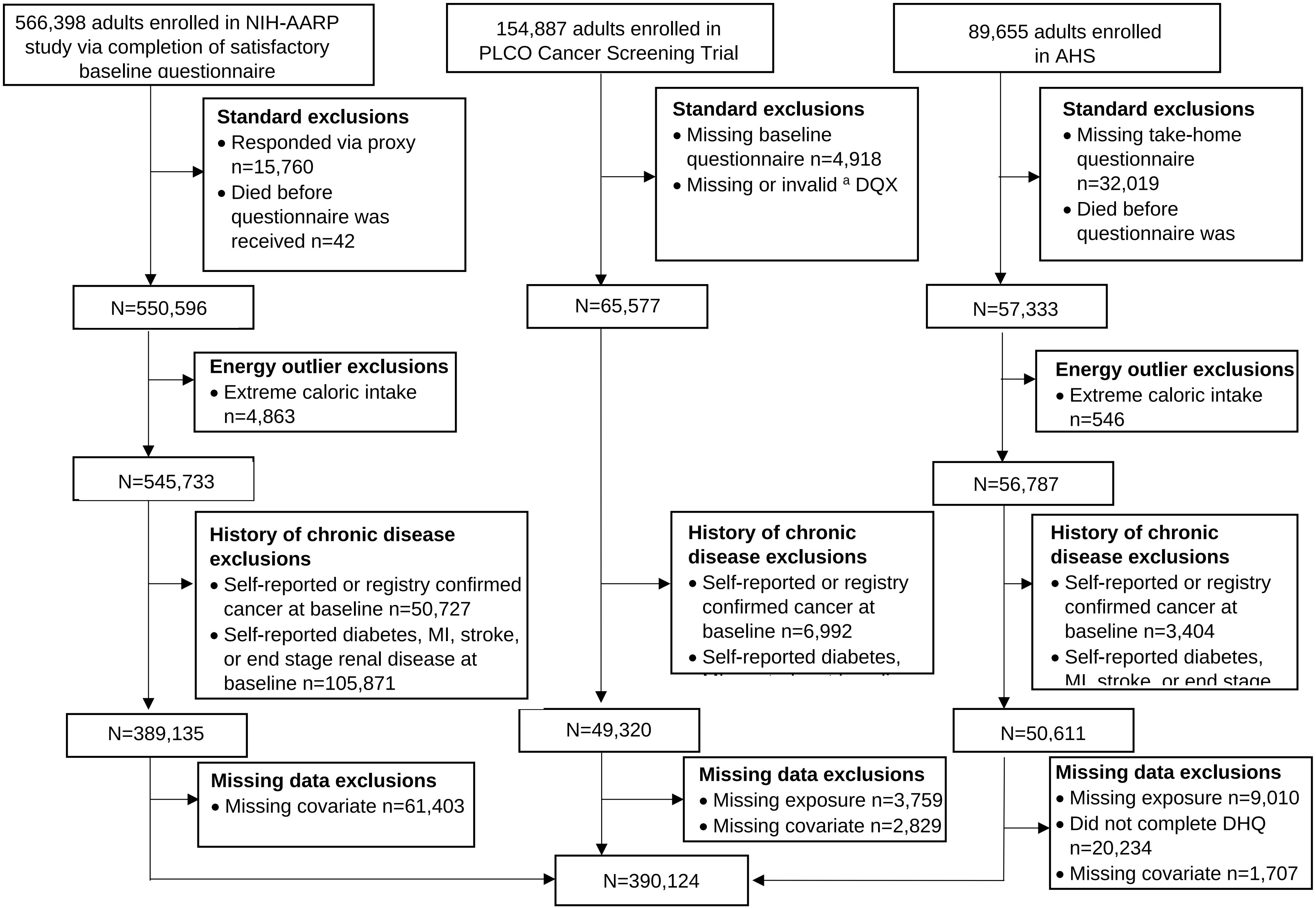


© 2024 Loftfield E et al. *JAMA Network Open.*

## eFigure 1. Flowchart for final analytic sample

[a] A Dietary Questionnaire (DQX) was considered invalid if the participant died before completion, had more than 8 missing or multiple-frequency responses, had a missing completion date or had extreme calorie consumption.

© 2024 Loftfield E et al. *JAMA Network Open.*

## eTable 1. Baseline characteristics of study participants by cohort, according to multivitamin use

| Characteristic, No. (%) [a] | Nonuse | Nondaily use | Daily use | Overall |
|---|---|---|---|---|
| **AARP participants** | **N=144947** | **N=41829** | **N=140956** | **N=327732** |
| Age, median (IQR), y | 62.0 (57.2 - 66.2) | 60.7 (56.2 - 65.2) | 62.2 (57.4 - 66.3) | 61.9 (57.2 - 66.1) |
| Sex | | | | |
| Female | 55143 (38.0%) | 19723 (47.2%) | 66615 (47.3%) | 141481 (43.2%) |
| Male | 89804 (62.0%) | 22106 (52.8%) | 74341 (52.7%) | 186251 (56.8%) |
| Race/Ethnicity | | | | |
| American Indian/Alaskan Native | 362 (0.2%) | 114 (0.3%) | 324 (0.2%) | 800 (0.2%) |
| Asian/Pacific Islander | 1897 (1.3%) | 729 (1.7%) | 1832 (1.3%) | 4458 (1.4%) |
| Hispanic | 2601 (1.8%) | 855 (2.0%) | 2371 (1.7%) | 5827 (1.8%) |
| Non-Hispanic Black | 5101 (3.5%) | 1843 (4.4%) | 3931 (2.8%) | 10875 (3.3%) |
| Non-Hispanic White | 134986 (93.1%) | 38288 (91.5%) | 132498 (94.0%) | 305772 (93.3%) |
| Education | | | | |
| less than 8 years, 8-11 years | 8612 (5.9%) | 1731 (4.1%) | 5999 (4.3%) | 16342 (5.0%) |
| 12 years, completed high school, GED | 30566 (21.1%) | 6987 (16.7%) | 26094 (18.5%) | 63647 (19.4%) |
| post-high school training | 14910 (10.3%) | 4171 (10.0%) | 13964 (9.9%) | 33045 (10.1%) |
| some college | 33294 (23.0%) | 10475 (25.0%) | 34327 (24.4%) | 78096 (23.8%) |
| college and postgraduate | 57565 (39.7%) | 18465 (44.1%) | 60572 (43.0%) | 136602 (41.7%) |
| Smoking status | | | | |
| Never smoker | 54867 (37.9%) | 16847 (40.3%) | 54369 (38.6%) | 126083 (38.5%) |
| Former smoker | 70391 (48.6%) | 19271 (46.1%) | 70506 (50.0%) | 160168 (48.9%) |
| Current smoker | 19689 (13.6%) | 5711 (13.7%) | 16081 (11.4%) | 41481 (12.7%) |
| Physical activity level | | | | |
| Never/Rarely | 27309 (18.8%) | 6437 (15.4%) | 20777 (14.7%) | 54523 (16.6%) |
| Low (few times a month) | 21516 (14.8%) | 6973 (16.7%) | 17804 (12.6%) | 46293 (14.1%) |
| Moderate (few times a week) | 32581 (22.5%) | 10848 (25.9%) | 30416 (21.6%) | 73845 (22.5%) |
| High (many times a week) | 63541 (43.8%) | 17571 (42.0%) | 71959 (51.1%) | 153071 (46.7%) |
| Individual supplement use | 50977 (35.2%) | 34005 (81.3%) | 119735 (84.9%) | 204717 (62.5%) |
| BMI category | | | | |
| < 18.5 kg/m2 | 1421 (1.0%) | 454 (1.1%) | 1685 (1.2%) | 3560 (1.1%) |
| 18.5 to < 25 kg/m2 | 49798 (34.4%) | 15558 (37.2%) | 56361 (40.0%) | 121717 (37.1%) |
| 25 to < 30 kg/m2 | 63020 (43.5%) | 17639 (42.2%) | 58095 (41.2%) | 138754 (42.3%) |
| ≥ 30 kg/m2 | 30708 (21.2%) | 8178 (19.6%) | 24815 (17.6%) | 63701 (19.4%) |
| Alcohol category | | | | |
| 0 drinks per day | 32001 (22.1%) | 7829 (18.7%) | 30147 (21.4%) | 69977 (21.4%) |
| Less than 1 drink per day | 76670 (52.9%) | 24509 (58.6%) | 77338 (54.9%) | 178517 (54.5%) |
| 1-2 drinks per day | 18147 (12.5%) | 4988 (11.9%) | 17730 (12.6%) | 40865 (12.5%) |
| 2-3 drinks per day | 5755 (4.0%) | 1510 (3.6%) | 5535 (3.9%) | 12800 (3.9%) |
| 3 or more drinks per day | 12374 (8.5%) | 2993 (7.2%) | 10206 (7.2%) | 25573 (7.8%) |
| Coffee intake | | | | |
| None | 15380 (10.6%) | 3920 (9.4%) | 15509 (11.0%) | 34809 (10.6%) |
| Less than 1 cup per day | 21837 (15.1%) | 7065 (16.9%) | 23029 (16.3%) | 51931 (15.8%) |
| 1 cup per day | 22996 (15.9%) | 6190 (14.8%) | 23186 (16.4%) | 52372 (16.0%) |
| 2-3 cups per day | 60156 (41.5%) | 17598 (42.1%) | 57639 (40.9%) | 135393 (41.3%) |
| 4-5 cups per day | 18685 (12.9%) | 5444 (13.0%) | 16650 (11.8%) | 40779 (12.4%) |
| 6 or more cups per day | 5893 (4.1%) | 1612 (3.9%) | 4943 (3.5%) | 12448 (3.8%) |

© 2024 Loftfield E et al. *JAMA Network Open.*

## eTable 1. Baseline characteristics of study participants by cohort, according to multivitamin use (continued)

| Characteristic, No. (%) [a] | Nonuse | Nondaily use | Daily use | Overall |
|---|---|---|---|---|
| HEI-2015 quartile | | | | |
| Quartile 1 | 43563 (30.1%) | 10502 (25.1%) | 29369 (20.8%) | 83434 (25.5%) |
| Quartile 2 | 37355 (25.8%) | 10834 (25.9%) | 33620 (23.9%) | 81809 (25.0%) |
| Quartile 3 | 33827 (23.3%) | 10601 (25.3%) | 36995 (26.2%) | 81423 (24.8%) |
| Quartile 4 | 30202 (20.8%) | 9892 (23.6%) | 40972 (29.1%) | 81066 (24.7%) |
| Marital status | | | | |
| Married or living as married | 104174 (71.9%) | 27806 (66.5%) | 92912 (65.9%) | 224892 (68.6%) |
| Divorced or separated | 19786 (13.7%) | 7658 (18.3%) | 24134 (17.1%) | 51578 (15.7%) |
| Widowed | 14059 (9.7%) | 4353 (10.4%) | 16555 (11.7%) | 34967 (10.7%) |
| Never married | 6928 (4.8%) | 2012 (4.8%) | 7355 (5.2%) | 16295 (5.0%) |
| Family history of cancer | 74673 (51.5%) | 21758 (52.0%) | 73402 (52.1%) | 169833 (51.8%) |
| Type of multivitamin | | | | |
| Stress- tab/B-complex/Other type | 0 (0%) | 2822 (6.7%) | 6239 (4.4%) | 9061 (2.8%) |
| Therapeutic or Theragran type | 0 (0%) | 5824 (13.9%) | 21726 (15.4%) | 27550 (8.4%) |
| One-a-day type | 0 (0%) | 30287 (72.4%) | 95962 (68.1%) | 126249 (38.5%) |
| More than 1 type | 0 (0%) | 2896 (6.9%) | 17029 (12.1%) | 19925 (6.1%) |
| **PLCO participants** | **N=19353** | **N=4471** | **N=18908** | **N=42732** |
| Age, median (IQR), y | 62.0 (58.0 - 66.0) | 61.0 (57.0 - 65.0) | 62.0 (58.0 - 66.0) | 62.0 (58.0 - 66.0) |
| Sex | | | | |
| Female | 8187 (42.3%) | 2503 (56.0%) | 11414 (60.4%) | 22104 (51.7%) |
| Male | 11166 (57.7%) | 1968 (44.0%) | 7494 (39.6%) | 20628 (48.3%) |
| Race/Ethnicity [b] | | | | |
| American Indian/Alaskan Native | 33 (0.2%) | 13 (0.3%) | 36 (0.2%) | 82 (0.2%) |
| Asian/Pacific Islander | 599 (3.1%) | 175 (3.9%) | 674 (3.6%) | 1448 (3.4%) |
| Hispanic | 241 (1.2%) | 59 (1.3%) | 267 (1.4%) | 567 (1.3%) |
| Non-Hispanic Black | 676 (3.5%) | 135 (3.0%) | 500 (2.6%) | 1311 (3.1%) |
| Non-Hispanic White | 17804 (92.0%) | 4089 (91.5%) | 17431 (92.2%) | 39324 (92.0%) |
| Education | | | | |
| less than 8 years, 8-11 years | 1322 (6.8%) | 173 (3.9%) | 949 (5.0%) | 2444 (5.7%) |
| 12 years, completed high school, GED | 4735 (24.5%) | 821 (18.4%) | 4236 (22.4%) | 9792 (22.9%) |
| post-high school training | 2411 (12.5%) | 529 (11.8%) | 2372 (12.5%) | 5312 (12.4%) |
| some college | 3948 (20.4%) | 991 (22.2%) | 4176 (22.1%) | 9115 (21.3%) |
| college and postgraduate | 6937 (35.8%) | 1957 (43.8%) | 7175 (37.9%) | 16069 (37.6%) |
| Smoking status | | | | |
| Never smoker | 9114 (47.1%) | 2284 (51.1%) | 9321 (49.3%) | 20719 (48.5%) |
| Former smoker | 8177 (42.3%) | 1754 (39.2%) | 7963 (42.1%) | 17894 (41.9%) |
| Current smoker | 2062 (10.7%) | 433 (9.7%) | 1624 (8.6%) | 4119 (9.6%) |
| Physical activity level | | | | |
| Never/Rarely | 3245 (16.8%) | 466 (10.4%) | 2420 (12.8%) | 6131 (14.3%) |
| Low (few times a month) | 3660 (18.9%) | 874 (19.5%) | 3108 (16.4%) | 7642 (17.9%) |
| Moderate (few times a week) | 5366 (27.7%) | 1358 (30.4%) | 5387 (28.5%) | 12111 (28.3%) |
| High (many times a week) | 7082 (36.6%) | 1773 (39.7%) | 7993 (42.3%) | 16848 (39.4%) |
| Individual supplement use | 6554 (33.9%) | 3638 (81.4%) | 15398 (81.4%) | 25590 (59.9%) |

© 2024 Loftfield E et al. *JAMA Network Open.*

eTable 1. Baseline characteristics of study participants by cohort, according to multivitamin use (continued)

| Characteristic, No. (%) [a] | Nonuse | Nondaily use | Daily use | Overall |
|---|---|---|---|---|
| BMI category | | | | |
| < 18.5 kg/m2 | 105 (0.5%) | 31 (0.7%) | 159 (0.8%) | 295 (0.7%) |
| 18.5 to < 25 kg/m2 | 6111 (31.6%) | 1751 (39.2%) | 7255 (38.4%) | 15117 (35.4%) |
| 25 to < 30 kg/m2 | 8682 (44.9%) | 1817 (40.6%) | 7713 (40.8%) | 18212 (42.6%) |
| ≥ 30 kg/m2 | 4455 (23.0%) | 872 (19.5%) | 3781 (20.0%) | 9108 (21.3%) |
| Alcohol category | | | | |
| 0 drinks per day | 3810 (19.7%) | 878 (19.6%) | 4188 (22.1%) | 8876 (20.8%) |
| Less than 1 drink per day | 10369 (53.6%) | 2553 (57.1%) | 10511 (55.6%) | 23433 (54.8%) |
| 1-2 drinks per day | 2322 (12.0%) | 479 (10.7%) | 2052 (10.9%) | 4853 (11.4%) |
| 2-3 drinks per day | 889 (4.6%) | 207 (4.6%) | 791 (4.2%) | 1887 (4.4%) |
| 3 or more drinks per day | 1963 (10.1%) | 354 (7.9%) | 1366 (7.2%) | 3683 (8.6%) |
| Coffee intake | | | | |
| None | 2106 (10.9%) | 572 (12.8%) | 2398 (12.7%) | 5076 (11.9%) |
| Less than 1 cup per day | 3724 (19.2%) | 967 (21.6%) | 3867 (20.5%) | 8558 (20.0%) |
| 1 cup per day | 4225 (21.8%) | 856 (19.1%) | 4195 (22.2%) | 9276 (21.7%) |
| 2-3 cups per day | 5934 (30.7%) | 1330 (29.7%) | 5622 (29.7%) | 12886 (30.2%) |
| 4-5 cups per day | 2172 (11.2%) | 528 (11.8%) | 1947 (10.3%) | 4647 (10.9%) |
| 6 or more cups per day | 1192 (6.2%) | 218 (4.9%) | 879 (4.6%) | 2289 (5.4%) |
| HEI-2015 quartile | | | | |
| Quartile 1 | 5880 (30.4%) | 969 (21.7%) | 3713 (19.6%) | 10562 (24.7%) |
| Quartile 2 | 5193 (26.8%) | 1101 (24.6%) | 4489 (23.7%) | 10783 (25.2%) |
| Quartile 3 | 4539 (23.5%) | 1156 (25.9%) | 5090 (26.9%) | 10785 (25.2%) |
| Quartile 4 | 3741 (19.3%) | 1245 (27.8%) | 5616 (29.7%) | 10602 (24.8%) |
| Marital status | | | | |
| Married or living as married | 15757 (81.4%) | 3378 (75.6%) | 14455 (76.4%) | 33590 (78.6%) |
| Divorced or separated | 1720 (8.9%) | 598 (13.4%) | 2133 (11.3%) | 4451 (10.4%) |
| Widowed | 1297 (6.7%) | 338 (7.6%) | 1702 (9.0%) | 3337 (7.8%) |
| Never married | 579 (3.0%) | 157 (3.5%) | 618 (3.3%) | 1354 (3.2%) |
| Family history of cancer | 10798 (55.8%) | 2541 (56.8%) | 10760 (56.9%) | 24099 (56.4%) |
| Type of multivitamin | | | | |
| Stress- tab/B-complex/Other type | 0 (0%) | 926 (20.7%) | 3171 (16.8%) | 4097 (9.6%) |
| Therapeutic or Theragran type | 0 (0%) | 276 (6.2%) | 1611 (8.5%) | 1887 (4.4%) |
| One-a-day type | 0 (0%) | 1861 (41.6%) | 9739 (51.5%) | 11600 (27.1%) |
| More than 1 type | 0 (0%) | 1408 (31.5%) | 4387 (23.2%) | 5795 (13.6%) |
| **AHS participants** | **N=15366** | **N=851** | **N=3443** | **N=19660** |
| Age, median (IQR), y | 47.0 (39.0 - 56.0) | 45.0 (37.0 - 55.0) | 48.0 (38.0 - 57.0) | 47.0 (38.0 - 56.0) |
| Sex | | | | |
| Female | 7298 (47.5%) | 549 (64.5%) | 2490 (72.3%) | 10337 (52.6%) |
| Male | 8068 (52.5%) | 302 (35.5%) | 953 (27.7%) | 9323 (47.4%) |
| Race/Ethnicity [b] | | | | |
| American Indian/Alaskan Native | 22 (0.1%) | 1 (0.1%) | 3 (0.1%) | 26 (0.1%) |
| Asian/Pacific Islander | 4 (0.0%) | 0 (0.0%) | 1 (0.0%) | 5 (0.0%) |
| Hispanic | 115 (0.7%) | 5 (0.7%) | 19 (0.6%) | 139 (0.7%) |
| Non-Hispanic Black | 66 (0.4%) | 2 (0.3%) | 9 (0.3%) | 77 (0.4%) |
| Non-Hispanic White | 15159 (98.7%) | 843 (99.1%) | 3411 (99.1%) | 19413 (98.7%) |

© 2024 Loftfield E et al. *JAMA Network Open.*

## eTable 1. Baseline characteristics of study participants by cohort, according to multivitamin use (continued)

| Characteristic, No. (%) [a] | Nonuse | Nondaily use | Daily use | Overall |
|---|---|---|---|---|
| Education | | | | |
| less than 8 years, 8-11 years | 677 (4.4%) | 21 (2.5%) | 116 (3.4%) | 814 (4.1%) |
| 12 years, completed high school, GED | 6515 (42.4%) | 261 (30.7%) | 1211 (35.2%) | 7987 (40.6%) |
| post-high school training | 1735 (11.3%) | 124 (14.6%) | 409 (11.9%) | 2268 (11.5%) |
| some college | 2377 (15.5%) | 156 (18.3%) | 587 (17.0%) | 3120 (15.9%) |
| college and postgraduate | 3530 (23.0%) | 237 (27.8%) | 881 (25.6%) | 4648 (23.6%) |
| other | 532 (3.5%) | 52 (6.1%) | 239 (6.9%) | 823 (4.2%) |
| Smoking status | | | | |
| Never smoker | 9862 (64.2%) | 600 (70.5%) | 2428 (70.5%) | 12890 (65.6%) |
| Former smoker | 3855 (25.1%) | 182 (21.4%) | 794 (23.1%) | 4831 (24.6%) |
| Current smoker | 1649 (10.7%) | 69 (8.1%) | 221 (6.4%) | 1939 (9.9%) |
| Physical activity level | | | | |
| Never/Rarely | 3265 (21.2%) | 119 (14.0%) | 569 (16.5%) | 3953 (20.1%) |
| Low (few times a month) | 2797 (18.2%) | 166 (19.5%) | 616 (17.9%) | 3579 (18.2%) |
| Moderate (few times a week) | 2816 (18.3%) | 181 (21.3%) | 687 (20.0%) | 3684 (18.7%) |
| High (many times a week) | 6488 (42.2%) | 385 (45.2%) | 1571 (45.6%) | 8444 (43.0%) |
| Individual supplement use | 5044 (32.8%) | 505 (59.3%) | 2037 (59.2%) | 7586 (38.6%) |
| BMI category | | | | |
| < 18.5 kg/m2 | 101 (0.7%) | 12 (1.4%) | 50 (1.5%) | 163 (0.8%) |
| 18.5 to < 25 kg/$m^2$ | 5462 (35.5%) | 389 (45.7%) | 1541 (44.8%) | 7392 (37.6%) |
| 25 to < 30 kg/$m^2$ | 6514 (42.4%) | 312 (36.7%) | 1320 (38.3%) | 8146 (41.4%) |
| ≥ 30 kg/$m^2$ | 3289 (21.4%) | 138 (16.2%) | 532 (15.5%) | 3959 (20.1%) |
| Alcohol category | | | | |
| 0 drinks per day | 5504 (35.8%) | 278 (32.7%) | 1310 (38.0%) | 7092 (36.1%) |
| Less than 1 drink per day | 8677 (56.5%) | 519 (61.0%) | 1962 (57.0%) | 11158 (56.8%) |
| 1-2 drinks per day | 851 (5.5%) | 40 (4.7%) | 134 (3.9%) | 1025 (5.2%) |
| 2-3 drinks per day | 244 (1.6%) | 10 (1.2%) | 23 (0.7%) | 277 (1.4%) |
| 3 or more drinks per day | 90 (0.6%) | 4 (0.5%) | 14 (0.4%) | 108 (0.5%) |
| Coffee intake | | | | |
| None | 4543 (29.6%) | 253 (29.7%) | 1086 (31.5%) | 5882 (29.9%) |
| Less than 1 cup per day | 3140 (20.4%) | 195 (22.9%) | 720 (20.9%) | 4055 (20.6%) |
| 1 cup per day | 2016 (13.1%) | 100 (11.8%) | 443 (12.9%) | 2559 (13.0%) |
| 2-3 cups per day | 3990 (26.0%) | 214 (25.1%) | 846 (24.6%) | 5050 (25.7%) |
| 4-5 cups per day | 1226 (8.0%) | 68 (8.0%) | 258 (7.5%) | 1552 (7.9%) |
| 6 or more cups per day | 451 (2.9%) | 21 (2.5%) | 90 (2.6%) | 562 (2.9%) |
| HEI-2015 quartile | | | | |
| Quartile 1 | 7713 (50.2%) | 353 (41.5%) | 1239 (36.0%) | 9305 (47.3%) |
| Quartile 2 | 4189 (27.3%) | 251 (29.5%) | 1047 (30.4%) | 5487 (27.9%) |
| Quartile 3 | 2282 (14.9%) | 151 (17.7%) | 692 (20.1%) | 3125 (15.9%) |
| Quartile 4 | 1182 (7.7%) | 96 (11.3%) | 465 (13.5%) | 1743 (8.9%) |
| Marital status | | | | |
| Married or living as married | 14262 (92.8%) | 792 (93.1%) | 3296 (95.7%) | 18350 (93.3%) |
| Divorced or separated | 245 (1.6%) | 20 (2.4%) | 32 (0.9%) | 297 (1.5%) |
| Widowed | 77 (0.5%) | 6 (0.7%) | 12 (0.3%) | 95 (0.5%) |
| Never married | 782 (5.1%) | 33 (3.9%) | 103 (3.0%) | 918 (4.7%) |
| Family history of cancer | 7431 (48.4%) | 410 (48.2%) | 1720 (50.0%) | 9561 (48.6%) |

© 2024 Loftfield E et al. *JAMA Network Open.*

## eTable 1. Baseline characteristics of study participants by cohort, according to multivitamin use (continued)

| Characteristic, No. (%) [a] | Nonuse | Nondaily use | Daily use | Overall |
|---|---|---|---|---|
| Type of multivitamin | | | | |
| Stress- tab/B-complex/Other | 0 (0%) | 84 (9.9%) | 219 (6.4%) | 303 (1.5%) |
| Therapeutic or Theragran | 0 (0%) | 63 (7.4%) | 266 (7.7%) | 329 (1.7%) |
| One-a-day | 0 (0%) | 684 (80.4%) | 2826 (82.1%) | 3510 (17.9%) |
| More than 1 type | 0 (0%) | 20 (2.4%) | 132 (3.8%) | 152 (0.8%) |

Abbreviations: HEI-2015 – Healthy Eating Index 2015; BMI – Body Mass Index

[a] Percentages are column percentages.

[b] Race or ethnic group was self-reported.

© 2024 Loftfield E et al. *JAMA Network Open.*

## eTable 2. Study-specific hazard ratios between multivitamin use and mortality (all-cause and cause-specific)

| Cause of Death | NIH-AARP | | | | |
|---|---|---|---|---|---|
| | Follow-up period 1[a] | | Follow-up period 2[a] | | *P* value for trend |
| **All-cause mortality** | **Nondaily use** | **Daily use** | **Nondaily** | **Daily** | |
| N deaths | 4063 | 15980 | 12678 | 47269 | |
| Age and sex-adjusted HR (95%CI) | 1.06 (1.02-1.10) | 1.00 (0.98-1.02) | 0.92 (0.85-0.99) | 0.99 (0.94-1.04) | 0.58 |
| Multivariable-adjusted HR (95%CI)[b] | 1.09 (1.05-1.13) | 1.05 (1.02-1.07) | 0.94 (0.88-1.02) | 1.03 (0.98-1.08) | <.001 |
| **Heart disease mortality** | | | | | |
| N deaths | 706 | 3073 | 2701 | 10458 | |
| Age and sex-adjusted HR (95%CI) | 0.98 (0.90-1.06) | 1.00 (0.96-1.06) | 0.90 (0.76-1.07) | 0.99 (0.89-1.10) | 0.80 |
| Multivariable-adjusted HR (95%CI)[b] | 1.02 (0.94-1.11) | 1.06 (1.01-1.12) | 0.92 (0.78-1.10) | 1.03 (0.93-1.15) | 0.01 |
| **Cancer mortality** | | | | | |
| N deaths | 1761 | 6625 | 3454 | 11923 | |
| Age and sex-adjusted HR (95%CI) | 1.03 (0.98-1.09) | 0.96 (0.93-0.99) | 0.90 (0.80-1.01) | 0.94 (0.87-1.01) | 0.01 |
| Multivariable-adjusted HR (95%CI)[b] | 1.06 (1.01-1.12) | 1.02 (0.98-1.05) | 0.93 (0.83-1.05) | 0.98 (0.91-1.06) | 0.38 |
| **Cerebrovascular diseases mortality** | | | | | |
| N deaths | 162 | 723 | 761 | 3000 | |
| Age and sex-adjusted HR (95%CI) | 1.04 (0.87-1.23) | 1.07 (0.96-1.19) | 0.97 (0.68-1.39) | 1.07 (0.85-1.33) | 0.21 |
| Multivariable-adjusted HR (95%CI)[b] | 1.02 (0.86-1.21) | 1.06 (0.95-1.18) | 0.96 (0.67-1.37) | 1.05 (0.84-1.32) | 0.28 |

| Cause of Death | PLCO | | | | |
|---|---|---|---|---|---|
| | Follow-up period 1[a] | | Follow-up period 2[a] | | *P* value for trend |
| **All-cause mortality** | **Nondaily use** | **Daily use** | **Nondaily** | **Daily** | |
| N deaths | 548 | 2379 | 927 | 4495 | |
| Age and sex-adjusted HR (95%CI) | 0.96 (0.88-1.06) | 0.91 (0.86-0.96) | 0.92 (0.75-1.14) | 1.07 (0.95-1.21) | 0.14 |
| Multivariable-adjusted HR (95%CI)[b] | 1.00 (0.91-1.10) | 0.95 (0.90-1.01) | 0.95 (0.77-1.18) | 1.10 (0.97-1.25) | 0.99 |
| **Heart disease mortality** | | | | | |
| N deaths | 104 | 428 | 188 | 958 | |
| Age and sex-adjusted HR (95%CI) | 1.04 (0.84-1.29) | 0.93 (0.82-1.06) | 0.91 (0.57-1.47) | 1.10 (0.83-1.47) | 0.69 |
| Multivariable-adjusted HR (95%CI)[b] | 1.13 (0.91-1.40) | 0.99 (0.86-1.14) | 0.96 (0.59-1.55) | 1.15 (0.86-1.54) | 0.65 |

© 2024 Loftfield E et al. *JAMA Network Open.*

## eTable 2. Study-specific hazard ratios between multivitamin use and mortality (all-cause and cause-specific) (continued)

| PLCO (continued) | Follow-up period 1[a] | | Follow-up period 2[a] | | *P* value for trend |
|---|---|---|---|---|---|
| **Cancer mortality** | | | | | |
| N deaths | 211 | 955 | 217 | 966 | |
| Age and sex-adjusted HR (95%CI) | 0.87 (0.75-1.01) | 0.89 (0.82-0.97) | 0.87 (0.61-1.24) | 0.96 (0.78-1.19) | 0.11 |
| Multivariable-adjusted HR (95%CI) [b] | 0.90 (0.77-1.04) | 0.93 (0.84-1.02) | 0.90 (0.63-1.29) | 0.99 (0.80-1.23) | 0.61 |
| **Cerebrovascular diseases mortality** | | | | | |
| N deaths | 27 | 117 | 69 | 304 | |
| Age and sex-adjusted HR (95%CI) | 0.97 (0.64-1.47) | 0.87 (0.68-1.13) | 1.11 (0.45-2.75) | 1.13 (0.65-1.96) | 0.26 |
| Multivariable-adjusted HR (95%CI) [b] | 0.99 (0.65-1.52) | 0.91 (0.70-1.18) | 1.14 (0.45-2.84) | 1.14 (0.65-2.00) | 0.34 |

| Cause of Death | AHS | | | | |
|---|---|---|---|---|---|
| | Follow-up period 1[a] | | Follow-up period 2[a] | | *P* value for trend |
| **All-cause mortality** | **Nondaily use** | **Daily use** | **Nondaily** | **Daily** | |
| N deaths | 25 | 102 | 86 | 452 | |
| Age and sex-adjusted HR (95%CI) | 1.08 (0.72-1.63) | 0.97 (0.78-1.21) | 0.85 (0.36-2.02) | 1.00 (0.63-1.58) | 0.83 |
| Multivariable-adjusted HR (95%CI) [b] | 1.04 (0.69-1.56) | 1.18 (0.95-1.48) | 0.92 (0.38-2.21) | 1.08 (0.67-1.72) | 0.12 |
| **Heart disease mortality** | | | | | |
| N deaths | 3 | 27 | 17 | 87 | |
| Age and sex-adjusted HR (95%CI) | Did not converge | 1.12 (0.73-1.72) | Did not converge | 0.92 (0.37-2.30) | 0.53 |
| Multivariable-adjusted HR (95%CI) [b] | Did not converge | 1.54 (0.98-2.40) | Did not converge | 0.98 (0.38-2.51) | <.001 |
| **Cancer mortality** | | | | | |
| N deaths | 13 | 41 | 29 | 137 | |
| Age and sex-adjusted HR (95%CI) | 1.35 (0.77-2.38) | 0.99 (0.70-1.39) | 0.92 (0.27-3.20) | 1.03 (0.49-2.13) | 0.99 |
| Multivariable-adjusted HR (95%CI) [b] | 1.46 (0.82-2.60) | 1.17 (0.83-1.67) | 1.00 (0.28-3.55) | 1.10 (0.52-2.31) | 0.32 |
| **Cerebrovascular diseases mortality** | | | | | |
| N deaths | 1 | 10 | 5 | 24 | |
| Age and sex-adjusted HR (95%CI) | Did not converge | 1.69 (0.78-3.68) | Did not converge | 0.86 (0.16-4.51) | 0.002 |
| Multivariable-adjusted HR (95%CI) [b] | Did not converge | 2.16 (0.96-4.87) | Did not converge | 0.92 (0.16-5.14) | <.001 |

© 2024 Loftfield E et al. *JAMA Network Open.*

## eTable 2. Study-specific hazard ratios between multivitamin use and mortality (all-cause and cause-specific) (continued)

[a] The proportional hazards assumption was violated (*P* <.001). Therefore, follow-up time was stratified by the midpoint, and hazard ratios were calculated using an interaction term between follow-up period and the exposure variable. **NIH-AARP**: Maximum follow-up time was 24 years; follow-up period 1 was the first 12 years of follow-up and follow-up period 2 was the last 12 years of follow-up. **PLCO**: Maximum follow-up was 27 years; follow-up period 1 was the first 12 years of follow-up and follow-up period 2 was the last 15 years of follow-up. **AHS**: Maximum follow-up was 26 years; follow-up period 1 was the first 12 years of follow-up and follow-up period 2 was the last 14 years of follow-up.

[b] Models were adjusted for sex (male or female), age at enrollment (years), race/ethnicity (American Indian/Alaskan Native, Asian/Pacific Islander, Hispanic, Non-Hispanic Black, or Non-Hispanic White), education (11 years or less; 12 years, completed high school or GED; post high school training; some college; college and postgraduate; or other), body mass index category (< 18.5 $kg/m^2$, 18.5 to <25 $kg/m^2$, 25 to <30 $kg/m^2$, 30+ $kg/m^2$), marital status (married or living as married, divorced or separated, widowed, or never married), smoking status (never smoker, former smoker, current smoker ≤20 cigarettes/day, current smoker 21-40 cigarettes/day, or current smoker > 40 cigarettes/day), alcohol consumption (0 drinks per day, < 1 drink/day, 1 to <2 drinks/day, 2 to <3 drinks/day, 3 or more drinks/day), physical activity level (never, low: few times a month, moderate: few times per week, high: many times a week), coffee intake (0 cups/day, <1 cup/day, 2-3 cups/day, 4-5 cups/day, or 6+ cups/day), family history of cancer (yes or no), Healthy Eating Index 2015 (HEI-2015) quartile (**NIH-AARP**: Quartile 1: 21.5 to <61.5, Quartile 2: 61.5 to <68.6, Quartile 3: 68.6 to <74.7, Quartile 4: 74.7 to 98; **PLCO**: Quartile 1: 28.5 to <60.8, Quartile 2: 60.8 to <67.3, Quartile 3: 67.3 to <73.1, Quartile 4: 73.1 to 95; **AHS**: Quartile 1: 21.9 to <55.3, Quartile 2: 55.3 to <61.8, Quartile 3: 61.8 to <68.2, Quartile 4: 68.2 to 95), and use of individual supplements (yes or no). CI denotes confidence intervals and numbers represent hazard ratios and 95% confidence intervals for daily multivitamin users and nondaily users in adjusted models.

© 2024 Loftfield E et al. *JAMA Network Open.*

| Variable | N deaths daily use/ N deaths nonuse | HR (95% CI) | *P* value |
|---|---|---|---|
| **Overall** | | | |
| F1 | 4505 / 19025 | 1.09 (1.05-1.13) | |
| F2 | 13822 / 56733 | 0.95 (0.88-1.02) | |
| **Sex** | | | 0.13 |
| Males | | | |
| F1 | 2752 / 13040 | 1.07 (1.03-1.11) | |
| F2 | 7816 / 36688 | 0.97 (0.88-1.06) | |
| Females | | | |
| F1 | 1753 / 5985 | 1.09 (1.04-1.15) | |
| F2 | 6006 / 20045 | 0.93 (0.83-1.05) | |
| **Age category** | | | <.001 |
| Under 55 years | | | |
| F1 | 335 / 1084 | 0.97 (0.85-1.10) | |
| F2 | 1305 / 4583 | 0.93 (0.71-1.21) | |
| 55 - 59 years | | | |
| F1 | 800 / 2903 | 1.09 (1.01-1.18) | |
| F2 | 2805 / 9779 | 0.97 (0.82-1.15) | |
| 60 - 64 years | | | |
| F1 | 1302 / 5196 | 1.03 (0.96-1.09) | |
| F2 | 4164 / 16225 | 0.98 (0.86-1.12) | |
| 65 - 69 years | | | |
| F1 | 1783 / 8295 | 1.03 (0.98-1.09) | |
| F2 | 4867 / 22328 | 0.96 (0.86-1.07) | |
| 70 years and older | | | |
| F1 | 285 / 1547 | 1.10 (0.97-1.26) | |
| F2 | 681 / 3818 | 0.93 (0.70-1.23) | |
| **Race/Ethnicity** | | | 0.50 |
| American Indian/Alaskan Native | | | |
| F1 | 15 /52 | 1.50 (0.79-2.88) | |
| F2 | 41 / 117 | 1.21 (0.30-4.79) | |
| Asian/Pacific Islander | | | |
| F1 | 57 / 212 | 1.23 (0.90-1.67) | |
| F2 | 160 / 614 | 0.81 (0.42-1.57) | |
| Hispanic | | | |
| F1 | 72 / 247 | 0.91 (0.69-1.20) | |
| F2 | 237 / 829 | 0.98 (0.55-1.76) | |
| Non-Hispanic Black | | | |
| F1 | 182 / 700 | 1.04 (0.87-1.23) | |
| F2 | 601 / 1875 | 0.95 (0.66-1.36) | |
| Non-Hispanic White | | | |
| F1 | 4179 / 17814 | 1.09 (1.05-1.13) | |
| F2 | 12783 / 53298 | 0.95 (0.88-1.02) | |
| **Smoking status** | | | <.001 |
| Never Smokers | | | |
| F1 | 1241 / 4766 | 1.06 (1.00-1.13) | |
| F2 | 5010 / 19884 | 0.96 (0.84-1.09) | |
| Former Smokers | | | |
| F1 | 2022 / 9404 | 1.10 (1.05-1.16) | |
| F2 | 6478 / 27801 | 0.95 (0.85-1.05) | |
| Current Smokers | | | |
| F1 | 1242 / 4855 | 1.09 (1.02-1.16) | |
| F2 | 2334 / 9048 | 0.95 (0.83-1.10) | |
| **BMI category** | | | 0.001 |
| 18.5 to <25 kg/m2 | | | |
| F1 | 1694 / 6350 | 1.15 (1.09-1.22) | |
| F2 | 4914 / 18273 | 0.97 (0.86-1.09) | |
| 25 to <30 kg/m2 | | | |
| F1 | 1785 / 7851 | 1.05 (1.00-1.11) | |
| F2 | 5816 / 24791 | 0.94 (0.84-1.05) | |
| 30+ kg/m2 | | | |
| F1 | 936 / 4536 | 1.08 (1.00-1.16) | |
| F2 | 2944 / 13125 | 0.93 (0.80-1.09) | |
| **HEI-2015 quartile** | | | 0.49 |
| Quartile 1 | | | |
| F1 | 1371 / 6839 | 1.07 (1.01-1.13) | |
| F2 | 3389 / 16851 | 0.97 (0.85-1.10) | |
| Quartile 2 | | | |
| F1 | 1132 / 4917 | 1.09 (1.02-1.17) | |
| F2 | 3502 / 14591 | 0.96 (0.83-1.10) | |
| Quartile 3 | | | |
| F1 | 1080 / 4077 | 1.10 (1.03-1.18) | |
| F2 | 3491 / 13339 | 0.93 (0.80-1.07) | |
| Quartile 4 | | | |
| F1 | 922 / 3192 | 1.07 (0.99-1.15) | |
| F2 | 3440 / 11952 | 0.95 (0.81-1.11) | |

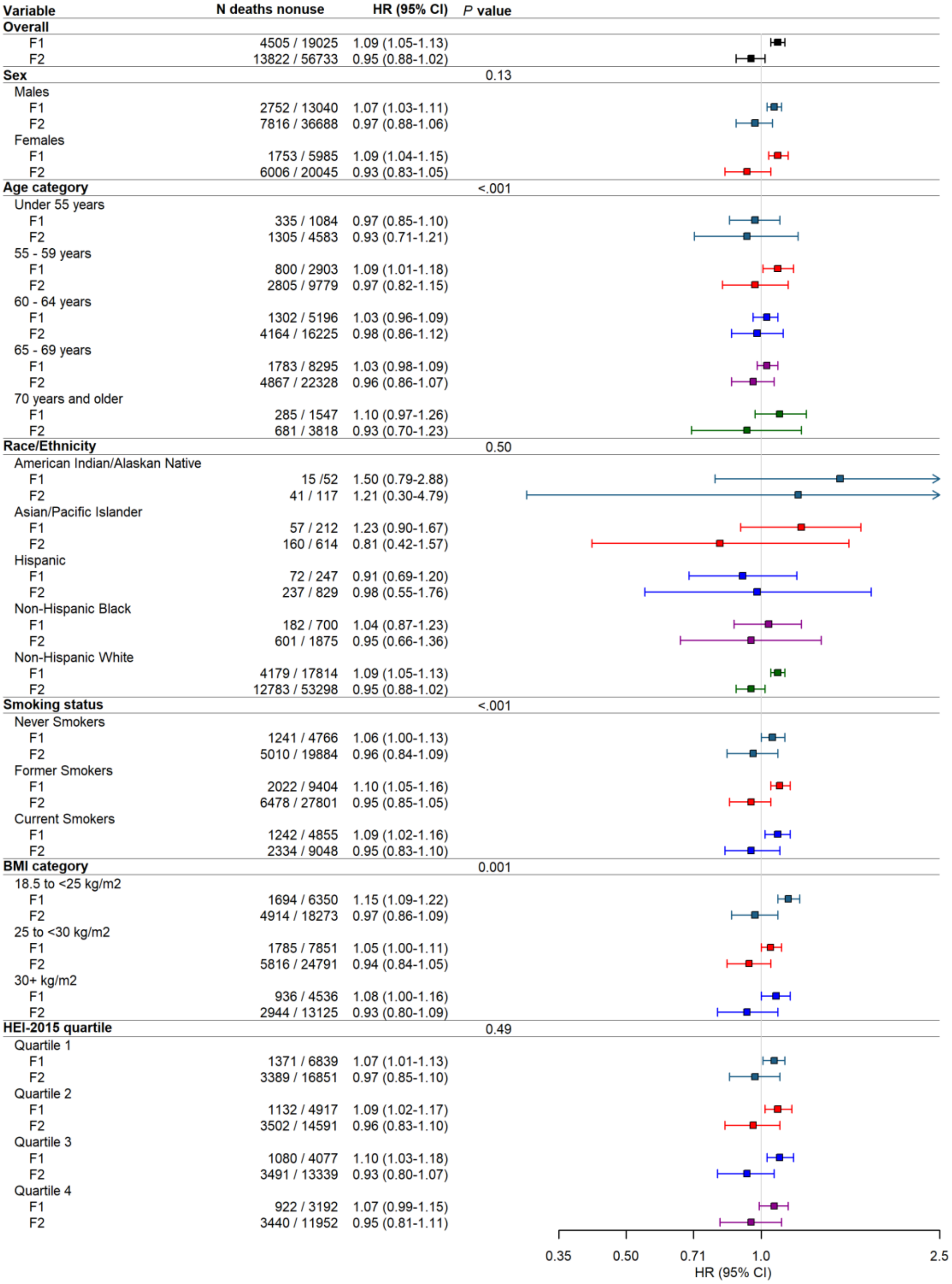


## eFigure 2. Forest plot of stratified baseline estimates for the association of nondaily multivitamin use and all-cause mortality, N=390,124

Abbreviations: Follow-up period 1 (F1); Follow-up period 2 (F2); Body Mass Index (BMI); Healthy Eating Index-2015 (HEI-2015)

The proportional hazards assumption was violated (*P*<.001). Therefore, follow-up time was stratified by the midpoint, and hazard ratios were calculated using an interaction term between follow-up period and the exposure variable. Maximum follow-up time was

© 2024 Loftfield E et al. *JAMA Network Open.*

24 years for the AARP cohort, 27 years for the PLCO cohort, and 26 years for the AHS cohort; follow-up period 1 (F1) was the first 12 years of follow-up and follow-up period 2 (F2) was the last 15 years of follow-up.

*P* value represents significance of likelihood ratio test for each effect modifier.

Models were stratified by study and adjusted for sex (male or female), age at enrollment (years), race/ethnicity (American Indian/Alaskan Native, Asian/Pacific Islander, Hispanic, Non-Hispanic Black, or Non-Hispanic White), education (11 years or less; 12 years, completed high school or GED; post high school training; some college; college and postgraduate; or other), body mass index category (< 18.5 kg/m2, 18.5 to <25 kg/m2, 25 to <30 kg/m2, 30+ kg/m2), marital status (married or living as married, divorced or separated, widowed, or never married), smoking status (never smoker, former smoker, current smoker ≤20 cigarettes/day, current smoker 21-40 cigarettes/day, or current smoker > 40 cigarettes/day), alcohol consumption (0 drinks per day, < 1 drink/day, 1 to <2 drinks/day, 2 to <3 drinks/day, 3 or more drinks/day), physical activity level (never, low: few times a month, moderate: few times per week, high: many times a week), coffee intake (0 cups/day, <1 cup/day, 2-3 cups/day, 4-5 cups/day, or 6+ cups/day), family history of cancer (yes or no), Healthy Eating Index 2015 (HEI-2015) quartile (Quartile 1: 21.55 to <60.90, Quartile 2: 60.90 to <68.00, Quartile 3: 68:00 to <74.20, Quartile 4: 74.20 to <96.10), and use of individual supplements (yes or no).

© 2024 Loftfield E et al. *JAMA Network Open.*

**eTable 3. Baseline characteristics of time-varying analysis participants by cohort, according to multivitamin use**

| Characteristic, No. (%) | Nonuse | Nondaily use | Daily use | *P* | Overall |
|---|---|---|---|---|---|
| **AARP participants** | **N=76010** | **N=21922** | **N=74564** | | **N=172496** |
| Age, median (IQR), y | 62.3 (57.9-66.7) | 60.9 (52.0-69.8) | 62.4 (53.6-71.2) | <.001 | 62.1 (57.7-66.5) |
| Sex | | | | <.001 | |
| Female | 28410 (37.4%) | 10294 (47.0%) | 35013 (47.0%) | | 73717 (42.7%) |
| Male | 47600 (62.6%) | 11628 (53.0%) | 39551 (53.0%) | | 98779 (57.3%) |
| Race/Ethnicity [a] | | | | <.001 | |
| American Indian/Alaskan Native | 144 (0.2%) | 48 (0.2%) | 146 (0.2%) | | 338 (0.2%) |
| Asian/Pacific Islander | 935 (1.2%) | 329 (1.5%) | 825 (1.1%) | | 2089 (1.2%) |
| Hispanic | 1152 (1.5%) | 400 (1.8%) | 1089 (1.5%) | | 2641 (1.5%) |
| Non-Hispanic Black | 2307 (3.0%) | 802 (3.7%) | 1780 (2.4%) | | 4889 (2.8%) |
| Non-Hispanic White | 71472 (94.0%) | 20343 (92.8%) | 70724 (94.9%) | | 162539 (94.2%) |
| Education | | | | <.001 | |
| less than 8 years, 8-11 years | 3569 (4.7%) | 728 (3.3%) | 2491 (3.3%) | | 6788 (3.9%) |
| 12 years, completed high school, GED | 14898 (19.6%) | 3417 (15.6%) | 12776 (17.1%) | | 31091 (18.0%) |
| post-high school training | 7625 (10.0%) | 2116 (9.7%) | 7220 (9.7%) | | 16961 (9.8%) |
| some college | 17175 (22.6%) | 5371 (24.5%) | 17850 (23.9%) | | 40396 (23.4%) |
| college and postgraduate | 32743 (43.1%) | 10290 (46.9%) | 34227 (45.9%) | | 77260 (44.8%) |
| Smoking status | | | | <.001 | |
| Never smoker | 29146 (38.3%) | 8931 (40.7%) | 28962 (38.8%) | | 67039 (38.9%) |
| Former smoker | 36995 (48.7%) | 10066 (45.9%) | 37214 (49.9%) | | 84275 (48.9%) |
| Current smoker | 9869 (13.0%) | 2925 (13.3%) | 8388 (11.2%) | | 21182 (12.3%) |
| Physical activity level | | | | <.001 | |
| Never/Rarely | 13905 (18.3%) | 3326 (15.2%) | 10858 (14.6%) | | 28089 (16.3%) |
| Low (few times a month) | 11311 (14.9%) | 3607 (16.5%) | 9436 (12.7%) | | 24354 (14.1%) |
| Moderate (few times per week) | 17211 (22.6%) | 5725 (26.1%) | 16164 (21.7%) | | 39100 (22.7%) |
| High (many times a week) | 33583 (44.2%) | 9264 (42.3%) | 38106 (51.1%) | | 80953 (46.9%) |
| Individual supplement use | 26754 (35.2%) | 17755 (81.0%) | 63151 (84.7%) | <.001 | 107660 (62.4%) |
| BMI category | | | | <.001 | |
| < 18.5 kg/m2 | 761 (1.0%) | 254 (1.2%) | 911 (1.2%) | | 1926 (1.1%) |
| 18.5 to < 25 kg/m2 | 26734 (35.2%) | 8306 (37.9%) | 30352 (40.7%) | | 65392 (37.9%) |
| 25 to < 30 kg/m2 | 32773 (43.1%) | 9183 (41.9%) | 30375 (40.7%) | | 72331 (41.9%) |
| ≥ 30 kg/m2 | 15742 (20.7%) | 4179 (19.1%) | 12926 (17.3%) | | 32847 (19.0%) |
| Alcohol category | | | | <.001 | |
| 0 drinks per day | 15708 (20.7%) | 3827 (17.5%) | 14958 (20.1%) | | 34493 (20.0%) |
| Less than 1 drink per day | 40429 (53.2%) | 12999 (59.3%) | 41428 (55.6%) | | 94856 (55.0%) |
| 1-2 drinks per day | 10025 (13.2%) | 2685 (12.2%) | 9658 (13.0%) | | 22368 (13.0%) |
| 2-3 drinks per day | 3128 (4.1%) | 804 (3.7%) | 3038 (4.1%) | | 6970 (4.0%) |
| 3 or more drinks per day | 6720 (8.8%) | 1607 (7.3%) | 5482 (7.4%) | | 13809 (8.0%) |
| Coffee intake, n (%) | | | | <.001 | |
| None | 8111 (10.7%) | 2056 (9.4%) | 8156 (10.9%) | | 18323 (10.6%) |
| Less than 1 cup per day | 11469 (15.1%) | 3592 (16.4%) | 12294 (16.5%) | | 27355 (15.9%) |
| 1 cup per day | 11907 (15.7%) | 3225 (14.7%) | 11990 (16.1%) | | 27122 (15.7%) |
| 2-3 cups per day | 31848 (41.9%) | 9330 (42.6%) | 30653 (41.1%) | | 71831 (41.6%) |
| 4-5 cups per day | 9730 (12.8%) | 2894 (13.2%) | 8882 (11.9%) | | 21506 (12.5%) |
| 6 or more cups per day | 2945 (3.9%) | 825 (3.8%) | 2589 (3.5%) | | 6359 (3.7%) |

© 2024 Loftfield E et al. *JAMA Network Open.*

## eTable 3. Baseline characteristics of time-varying analysis participants by cohort, according to multivitamin use (continued)

| Characteristic (AARP continued) | Nonuse | Nondaily use | Daily use | *P* | Overall |
|---|---|---|---|---|---|
| HEI-2015 quartile | | | | <.001 | |
| Quartile 1 | 22229 (29.2%) | 5374 (24.5%) | 15159 (20.3%) | | 42762 (24.8%) |
| Quartile 2 | 19581 (25.8%) | 5624 (25.7%) | 17746 (23.8%) | | 42951 (24.9%) |
| Quartile 3 | 18042 (23.7%) | 5593 (25.5%) | 19755 (26.5%) | | 43390 (25.2%) |
| Quartile 4 | 16158 (21.3%) | 5331 (24.3%) | 21904 (29.4%) | | 43393 (25.2%) |
| Marital status | | | | <.001 | |
| Married or living as married | 55052 (72.4%) | 14630 (66.7%) | 49106 (65.9%) | | 118788 (68.9%) |
| Divorced or separated | 9820 (12.9%) | 3897 (17.8%) | 12500 (16.8%) | | 26217 (15.2%) |
| Widowed | 7217 (9.5%) | 2268 (10.3%) | 8703 (11.7%) | | 18188 (10.5%) |
| Never married | 3921 (5.2%) | 1127 (5.1%) | 4255 (5.7%) | | 9303 (5.4%) |
| Family history of cancer | 39878 (52.5%) | 11554 (52.7%) | 39173 (52.5%) | <.001 | 90605 (52.5%) |
| **PLCO participants** | **N=19353** | **N=4471** | **N=18908** | | **N=42732** |
| Age, median (IQR), y | 62.0 (58.0 - 66.0) | 61.0 (57.0 - 65.0) | 62.0 (58.0 - 66.0) | <.001 | 62.0 (58.0 - 66.0) |
| Sex | | | | <.001 | |
| Female | 8187 (42.3%) | 2503 (56.0%) | 11414 (60.4%) | | 22104 (51.7%) |
| Male | 11166 (57.7%) | 1968 (44.0%) | 7494 (39.6%) | | 20628 (48.3%) |
| Race/Ethnicity [a] | | | | <.001 | |
| American Indian/Alaskan Native | 33 (0.2%) | 13 (0.3%) | 36 (0.2%) | | 82 (0.2%) |
| Asian/Pacific Islander | 599 (3.1%) | 175 (3.9%) | 674 (3.6%) | | 1448 (3.4%) |
| Hispanic | 241 (1.2%) | 59 (1.3%) | 267 (1.4%) | | 567 (1.3%) |
| Non-Hispanic Black | 676 (3.5%) | 135 (3.0%) | 500 (2.6%) | | 1311 (3.1%) |
| Non-Hispanic White | 17804 (92.0%) | 4089 (91.5%) | 17431 (92.2%) | | 39324 (92.0%) |
| Education | | | | <.001 | |
| less than 8 years, 8-11 years | 1322 (6.8%) | 173 (3.9%) | 949 (5.0%) | | 2444 (5.7%) |
| 12 years, completed high school, GED | 4735 (24.5%) | 821 (18.4%) | 4236 (22.4%) | | 9792 (22.9%) |
| post-high school training | 2411 (12.5%) | 529 (11.8%) | 2372 (12.5%) | | 5312 (12.4%) |
| some college | 3948 (20.4%) | 991 (22.2%) | 4176 (22.1%) | | 9115 (21.3%) |
| college and postgraduate | 6937 (35.8%) | 1957 (43.8%) | 7175 (37.9%) | | 16069 (37.6%) |
| Smoking status | | | | <.001 | |
| Never smoker | 9114 (47.1%) | 2284 (51.1%) | 9321 (49.3%) | | 20719 (48.5%) |
| Former smoker | 8177 (42.3%) | 1754 (39.2%) | 7963 (42.1%) | | 17894 (41.9%) |
| Current smoker | 2062 (10.7%) | 433 (9.7%) | 1624 (8.6%) | | 4119 (9.6%) |
| Physical activity level | | | | <.001 | |
| Never/Rarely | 3245 (16.8%) | 466 (10.4%) | 2420 (12.8%) | | 6131 (14.3%) |
| Low (few times a month) | 3660 (18.9%) | 874 (19.5%) | 3108 (16.4%) | | 7642 (17.9%) |
| Moderate (few times per week) | 5366 (27.7%) | 1358 (30.4%) | 5387 (28.5%) | | 12111 (28.3%) |
| High (many times a week) | 7082 (36.6%) | 1773 (39.7%) | 7993 (42.3%) | | 16848 (39.4%) |
| Individual supplement use | 6554 (33.9%) | 3638 (81.4%) | 15398 (81.4%) | <.001 | 25590 (59.9%) |
| BMI category | | | | <.001 | |
| < 18.5 kg/m2 | 105 (0.5%) | 31 (0.7%) | 159 (0.8%) | | 295 (0.7%) |
| 18.5 to < 25 kg/m2 | 6111 (31.6%) | 1751 (39.2%) | 7255 (38.4%) | | 15117 (35.4%) |
| 25 to < 30 kg/m2 | 8682 (44.9%) | 1817 (40.6%) | 7713 (40.8%) | | 18212 (42.6%) |
| ≥ 30 kg/m2 | 4455 (23.0%) | 872 (19.5%) | 3781 (20.0%) | | 9108 (21.3%) |

© 2024 Loftfield E et al. *JAMA Network Open.*

**eTable 3. Baseline characteristics of time-varying analysis participants by cohort, according to multivitamin use (continued)**

| Characteristic (PLCO continued) | Nonuse | Nondaily use | Daily use | *P* | Overall |
|---|---|---|---|---|---|
| Alcohol category | | | | <.001 | |
| 0 drinks per day | 3810 (19.7%) | 878 (19.6%) | 4188 (22.1%) | | 8876 (20.8%) |
| Less than 1 drink per day | 10369 (53.6%) | 2553 (57.1%) | 10511 (55.6%) | | 23433 (54.8%) |
| 1-2 drinks per day | 2322 (12.0%) | 479 (10.7%) | 2052 (10.9%) | | 4853 (11.4%) |
| 2-3 drinks per day | 889 (4.6%) | 207 (4.6%) | 791 (4.2%) | | 1887 (4.4%) |
| 3 or more drinks per day | 1963 (10.1%) | 354 (7.9%) | 1366 (7.2%) | | 3683 (8.6%) |
| Coffee intake | | | | <.001 | |
| None | 2106 (10.9%) | 572 (12.8%) | 2398 (12.7%) | | 5076 (11.9%) |
| Less than 1 cup per day | 3724 (19.2%) | 967 (21.6%) | 3867 (20.5%) | | 8558 (20.0%) |
| 1 cup per day | 4225 (21.8%) | 856 (19.1%) | 4195 (22.2%) | | 9276 (21.7%) |
| 2-3 cups per day | 5934 (30.7%) | 1330 (29.7%) | 5622 (29.7%) | | 12886 (30.2%) |
| 4-5 cups per day | 2172 (11.2%) | 528 (11.8%) | 1947 (10.3%) | | 4647 (10.9%) |
| 6 or more cups per day | 1192 (6.2%) | 218 (4.9%) | 879 (4.6%) | | 2289 (5.4%) |
| HEI-2015 quartile | | | | <.001 | |
| Quartile 1 | 5880 (30.4%) | 969 (21.7%) | 3713 (19.6%) | | 10562 (24.7%) |
| Quartile 2 | 5193 (26.8%) | 1101 (24.6%) | 4489 (23.7%) | | 10783 (25.2%) |
| Quartile 3 | 4539 (23.5%) | 1156 (25.9%) | 5090 (26.9%) | | 10785 (25.2%) |
| Quartile 4 | 3741 (19.3%) | 1245 (27.8%) | 5616 (29.7%) | | 10602 (24.8%) |
| Marital status | | | | <.001 | |
| Married or living as married | 15757 (81.4%) | 3378 (75.6%) | 14455 (76.4%) | | 33590 (78.6%) |
| Divorced or separated | 1720 (8.9%) | 598 (13.4%) | 2133 (11.3%) | | 4451 (10.4%) |
| Widowed | 1297 (6.7%) | 338 (7.6%) | 1702 (9.0%) | | 3337 (7.8%) |
| Never married | 579 (3.0%) | 157 (3.5%) | 618 (3.3%) | | 1354 (3.2%) |
| Family history of cancer | 10798 (55.8%) | 2541 (56.8%) | 10760 (56.9%) | 0.074 | 24099 (56.4%) |
| **AHS participants** | **N=15132** | **N=839** | **N=3394** | | **N=19365** |
| Age, median (IQR), y | 46.0 (38.0-56.0) | 45.0 (37.0-55.0) | 48.0 (38.0-57.0) | <.001 | 47.0 (38.0-56.0) |
| Sex | | | | <.001 | |
| Female | 7207 (47.6%) | 545 (65.0%) | 2458 (72.4%) | | 10210 (52.7%) |
| Male | 7925 (52.4%) | 294 (35.0%) | 936 (27.6%) | | 9155 (47.3%) |
| Race/Ethnicity [a] | | | | 0.46 | |
| American Indian/Alaskan Native | 4 (0.0%) | 0 (0%) | 3 (0.1%) | | 26 (0.1%) |
| Asian/Pacific Islander | 22 (0.1%) | 1 (0.1%) | 1 (0.0%) | | 5 (0.0%) |
| Hispanic | 112 (0.7%) | 5 (0.6%) | 19 (0.6%) | | 136 (0.7%) |
| Non-Hispanic Black | 64 (0.4%) | 2 (0.2%) | 9 (0.3%) | | 75 (0.4%) |
| Non-Hispanic White | 14930 (98.7%) | 831 (99.0%) | 3362 (99.1%) | | 19123 (98.8%) |
| Education | | | | <.001 | |
| less than 8 years, 8-11 years | 656 (4.3%) | 20 (2.4%) | 112 (3.3%) | | 788 (4.1%) |
| 12 years, completed high school, or GED | 6401 (42.3%) | 256 (30.5%) | 1194 (35.2%) | | 7851 (40.5%) |
| post-high school training | 1711 (11.3%) | 123 (14.7%) | 404 (11.9%) | | 2238 (11.6%) |
| Some college | 2352 (15.5%) | 154 (18.4%) | 577 (17.0%) | | 3083 (15.9%) |
| College and postgraduate | 3487 (23.0%) | 234 (27.9%) | 872 (25.7%) | | 4593 (23.7%) |
| Other | 525 (3.5%) | 52 (6.2%) | 235 (6.9%) | | 812 (4.2%) |
| Smoking status | | | | <.001 | |
| Never smoker | 9721 (64.2%) | 595 (70.9%) | 2398 (70.7%) | | 12714 (65.7%) |
| Former smoker | 3791 (25.1%) | 178 (21.2%) | 779 (23.0%) | | 4748 (24.5%) |
| Current smoker | 1620 (10.7%) | 66 (7.9%) | 217 (6.4%) | | 1903 (9.8%) |

© 2024 Loftfield E et al. *JAMA Network Open.*

**eTable 3. Baseline characteristics of time-varying analysis participants by cohort, according to multivitamin use (continued)**

| Characteristic (AHS continued) | Nonuse | Nondaily use | Daily use | *P* | Overall |
|---|---|---|---|---|---|
| Physical activity level | | | | <.001 | |
| Never | 3209 (21.2%) | 118 (14.1%) | 556 (16.4%) | | 3883 (20.1%) |
| Low (few times a month) | 2758 (18.2%) | 164 (19.5%) | 604 (17.8%) | | 3526 (18.2%) |
| Moderate (few times per week) | 2775 (18.3%) | 180 (21.5%) | 680 (20.0%) | | 3635 (18.8%) |
| High (many times a week) | 6390 (42.2%) | 377 (44.9%) | 1554 (45.8%) | | 8321 (43.0%) |
| Individual supplement use | 4970 (32.8%) | 499 (59.5%) | 2003 (59.0%) | <.001 | 7472 (38.6%) |
| BMI category | | | | <.001 | |
| < 18.5 kg/m2 | 100 (0.7%) | 12 (1.4%) | 50 (1.5%) | | 162 (0.8%) |
| 18.5 to < 25 kg/m2 | 5384 (35.6%) | 387 (46.1%) | 1522 (44.8%) | | 7293 (37.7%) |
| 25 to < 30 kg/m2 | 6415 (42.4%) | 302 (36.0%) | 1299 (38.3%) | | 8016 (41.4%) |
| ≥ 30 kg/m2 | 3233 (21.4%) | 138 (16.4%) | 523 (15.4%) | | 3894 (20.1%) |
| Alcohol category | | | | <.001 | |
| 0 drinks per day | 5410 (35.8%) | 276 (32.9%) | 1287 (37.9%) | | 6973 (36.0%) |
| Less than 1 drink per day | 8554 (56.5%) | 511 (60.9%) | 1940 (57.2%) | | 11005 (56.8%) |
| 1-2 drinks per day | 836 (5.5%) | 38 (4.5%) | 133 (3.9%) | | 1007 (5.2%) |
| 2-3 drinks per day | 242 (1.6%) | 10 (1.2%) | 22 (0.6%) | | 274 (1.4%) |
| 3 or more drinks per day | 90 (0.6%) | 4 (0.5%) | 12 (0.4%) | | 106 (0.5%) |
| Coffee intake | | | | 0.002 | |
| None | 4485 (29.6%) | 252 (30.0%) | 1076 (31.7%) | | 5813 (30.0%) |
| Less than 1 cup per day | 3099 (20.5%) | 194 (23.1%) | 713 (21.0%) | | 4006 (20.7%) |
| 1 cup per day | 1982 (13.1%) | 100 (11.9%) | 435 (12.8%) | | 2517 (13.0%) |
| 2-3 cups per day | 3912 (25.9%) | 206 (24.6%) | 828 (24.4%) | | 4946 (25.5%) |
| 4-5 cups per day | 1205 (8.0%) | 68 (8.1%) | 252 (7.4%) | | 1525 (7.9%) |
| 6 or more cups per day | 449 (3.0%) | 19 (2.3%) | 90 (2.7%) | | 558 (2.9%) |
| HEI-2015 quartile | | | | <.001 | |
| Quartile 1 | 4217 (27.9%) | 159 (19.0%) | 605 (17.8%) | | 4981 (25.7%) |
| Quartile 2 | 3942 (26.1%) | 222 (26.5%) | 742 (21.9%) | | 4906 (25.3%) |
| Quartile 3 | 3692 (24.4%) | 219 (26.1%) | 937 (27.6%) | | 4848 (25.0%) |
| Quartile 4 | 3281 (21.7%) | 239 (28.5%) | 1110 (32.7%) | | 4630 (23.9%) |
| Marital status | | | | <.001 | |
| Married or living as married | 14041 (92.8%) | 783 (93.3%) | 3248 (95.7%) | | 18072 (93.3%) |
| Divorced or separated | 239 (1.6%) | 19 (2.3%) | 32 (0.9%) | | 290 (1.5%) |
| Widowed | 75 (0.5%) | 6 (0.7%) | 12 (0.4%) | | 93 (0.5%) |
| Never married | 777 (5.1%) | 31 (3.7%) | 102 (3.0%) | | 910 (4.7%) |
| Family history of cancer | 7310 (48.3%) | 403 (48.0%) | 1694 (49.9%) | 0.071 | 9407 (48.6%) |

Abbreviations: HEI-2015 – Healthy Eating Index 2015; BMI – Body Mass Index

[a] Race or ethnic group was self-reported.

© 2024 Loftfield E et al. *JAMA Network Open.*
