# Supplementary material for: Multivitamin Use and Mortality Risk in 3 Prospective US Cohorts
Source: JAMA Netw Open. 2024 Jun 26;7(6):e2418729. doi: 10.1001/jamanetworkopen.2024.18729 (PMC11208972; doi:10.1001/jamanetworkopen.2024.18729)
Supplement: Supplement 2. — Data Sharing Statement [file jamanetwopen-e2418729-s002.pdf]

Data Sharing Statement

Loftfield. Multivitamin Use and Mortality Risk in 3 Prospective US Cohorts. *JAMA Netw Open*. Published June 26, 2024. doi:10.1001/jamanetworkopen.2024.18729

Data
Data available: Yes
Data types: Deidentified participant data, Data dictionary
How to access data: Data can be obtained on request. Requests should be directed to the NIH-AARP Diet and Health Study (https://dietandhealth.cancer.gov/), PLCO Trial (https://cdas.cancer.gov/plco/), and Agricultural Health Study (https://aghealth.nih.gov/collaboration/process.html), which all have a protocol for approving data requests. Study resources, including data dictionaries and information on questionnaires, are available at the study websites cited above.
When available: With publication

Supporting Documents
Document types: Statistical/analytic code
How to access documents: Requests for complete code can be sent to erikka.loftfield@nih.gov.
When available: With publication

Additional Information
Who can access the data: Data will be shared with researchers whose proposed use of the data has been approved
Types of analyses: Data will be shared for research purposes
Mechanisms of data availability: Data will be made available after approval of proposal and signed data access agreement
